# Using citizen science to expand the global map of landslides: Introducing the Cooperative Open Online Landslide Repository (COOLR)

**DOI:** 10.1371/journal.pone.0218657

**Published:** 2019-07-03

**Authors:** Caroline S. Juang, Thomas A. Stanley, Dalia B. Kirschbaum

**Affiliations:** 1 Hydrological Sciences Laboratory, NASA Goddard Space Flight Center, Greenbelt, MD, United States of America; 2 Hydrospheric and Biospheric Sciences, Science Systems and Applications, Inc., Lanham, MD, United States of America; 3 Universities Space Research Association, Columbia, MD, United States of America; Universitat Zurich Institut fur Volkswirtschaftslehre, SWITZERLAND

## Abstract

Robust inventories are vital for improving assessment of and response to deadly and costly landslide hazards. However, collecting landslide events in inventories is difficult at the global scale due to inconsistencies in or the absence of landslide reporting. Citizen science is a valuable opportunity for addressing some of these challenges. The new Cooperative Open Online Landslide Repository (COOLR) supplements data in a NASA-developed Global Landslide Catalog (GLC) with citizen science reports to build a more robust, publicly available global inventory. This manuscript introduces the COOLR project and its methods, evaluates the initial citizen science results from the first 13 months, and discusses future improvements to increase the global engagement with the project. The COOLR project (https://landslides.nasa.gov) contains Landslide Reporter, the first global citizen science project for landslides, and Landslide Viewer, a portal to visualize data from COOLR and other satellite and model products. From March 2018 to April 2019, 49 citizen scientists contributed 162 new landslide events to COOLR. These events spanned 37 countries in five continents. The initial results demonstrated that both expert and novice participants are contributing via Landslide Reporter. Citizen scientists are filling in data gaps through news sources in 11 different languages, in-person observations, and new landslide events occurring hundreds and thousands of kilometers away from any existing GLC data. The data is of sufficient accuracy to use in NASA susceptibility and hazard models. COOLR continues to expand as an open platform of landslide inventories with new data from citizen scientists, NASA scientists, and other landslide groups. Future work on the COOLR project will seek to increase participation and functionality of the platform as well as move towards collective post-disaster mapping.

## Introduction

Landslides, or mass movements, cause thousands of deaths and billions of dollars in infrastructural damage worldwide each year, warranting the need to understand their triggers and mitigate future losses [1,2]. Landslides are triggered by a variety of natural and human causes, most commonly rainfall but also earthquakes, freeze-thaw cycles, mining, and other causes. These triggers loosen slope materials, resulting in their downward and outward movement by gravity as landslides, debris flows, mudslides, rock falls, earthflows, and other mass movements—all referred to in this paper as landslides [3,4]. Knowledge of past landslides is the most important element for hazard and risk assessment because landslides are likely to occur in areas that have previously experienced a failure [5–7]. Therefore, the collection of past landslide information helps to predict future risk. To date, there are few global, publicly available inventories providing landslide data.

### The value and limitations of landslide inventories, and the Global Landslide Catalog (GLC)

**Fig 1:**
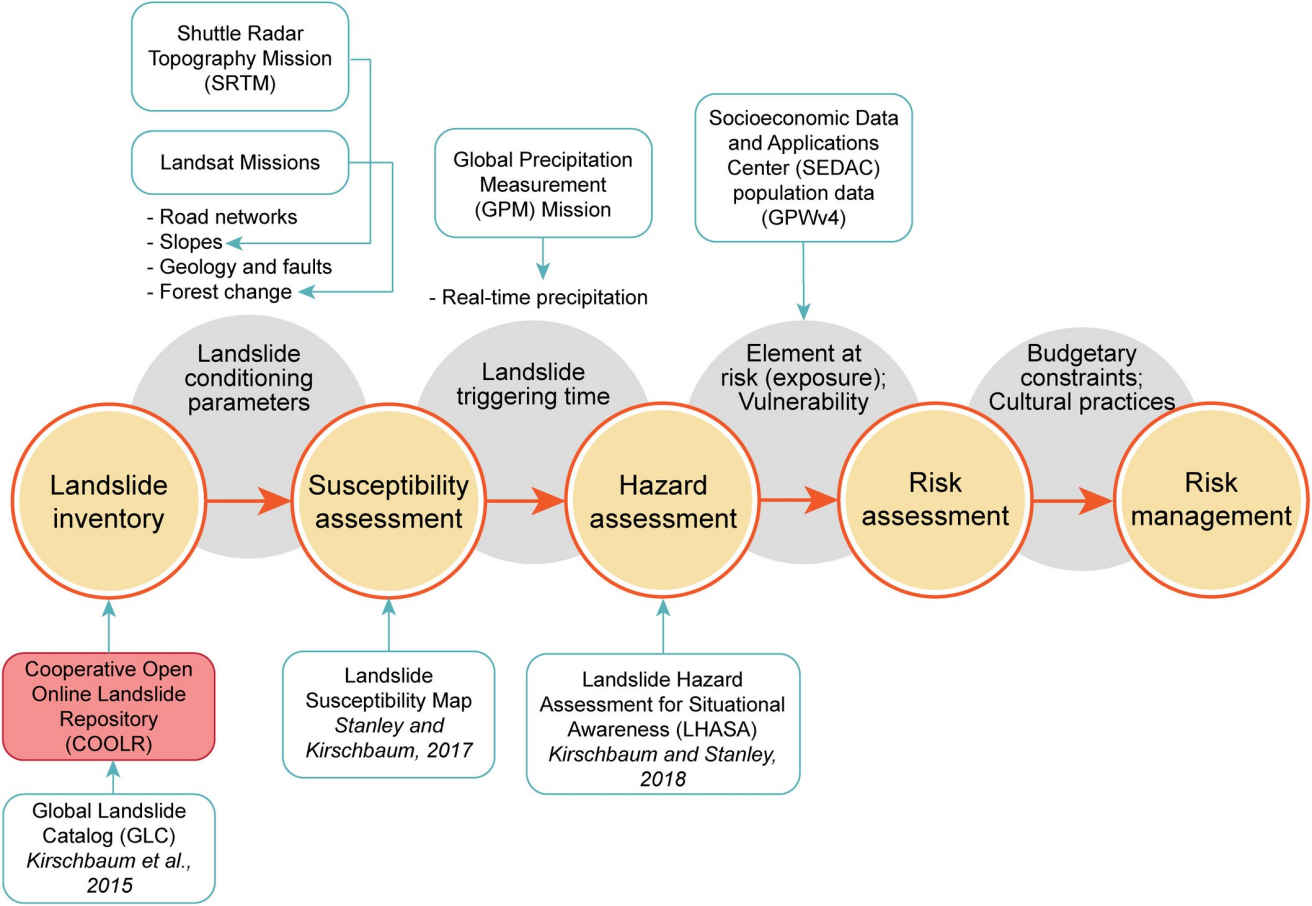
NASA’s role in landslide risk assessment. A simplified framework for how landslide inventories ultimately inform landslide risk assessment with the data inputs needed to reach each step, modified from Kocaman and Gokceoglu, 2018. NASA has developed several products that can support landslide risk assessment, outlined in teal. The contribution of a new NASA landslide inventory, the Cooperative Open Online Landslide Repository (COOLR), is shaded red.

Robust and complete landslide inventories are crucial for understanding past landslide mechanisms and forecasting future events. Landslide inventories, defined as spatial, temporal or spatiotemporal datasets of landslide events, are routinely used for many subject areas from characterizing landscape evolution, to calculating susceptibility and hazard, to supporting emergency response and planning efforts. There are different ways to compile a landslide inventory: collecting landslide distributions for a single triggering event; mapping landslides identified from satellite imagery or aerial photos, *in situ* mapping, or cataloging reports from news media [6,8–11]. Inventory production is the necessary first step for landslide risk management, but it must be followed by further work. NASA data and products help to support multiple steps in the process towards characterizing landslide risk (Fig 1), beginning with the landslide inventory.

The Global Landslide Catalog (GLC) exemplifies one type of landslide inventory that provides the estimated location and date of occurrence. The authors and supporting team members have been cataloging rainfall-triggered landslide events from 2007 to the present, collected primarily from online news reports found using a Google Alerts search for relevant keywords. A landslide event was defined as a single landslide or multiple landslides tied to a location associated with a single triggering event [12]. The GLC currently contains more than 11,500 reports, including 230 landslides from the Durham Fatal Landslide Database (DFLD) [12–14]. Gathering events from news reports was favorable to the authors’ scientific goals because news reports can give an accurate event date, tying the events to probable triggering conditions that can be used for model development [14]. In contrast, the dates of landslides cataloged from satellite or aerial imagery are uncertain due to typically long periods between image acquisitions. Information about landslides can be combined empirically or deterministically with data on precipitation, slope, lithology, surface cover, etc. to characterize or model patterns and trends in landslide behavior over short to long time scales and local to global areas [5,9,10,15,16]. On a global scale, this data can help infer patterns; for example, characterizing the seasonality of landslides in different regions of the world based on climate drivers [16], comparing areas of the world based on their vulnerability to landslides [2], and evaluating the distribution and cost of fatal landslides [13,17]. The GLC has been corroborated as a template for landslide inventory design [13,18] and as a validation tool for worldwide landslide hazard and susceptibility modeling [15,16,19,20].

Despite the need for comprehensive landslide catalogs, routine mapping of landslides is time-consuming and resource-intensive [8,21,22]. Unlike hurricanes or earthquakes that have global monitoring systems (e.g. Joint Typhoon Warning Center, http://www.metoc.navy.mil/jtwc/jtwc.html; World Meteorological Organization Tropical Cyclone Program, https://severe.worldweather.wmo.int/; Global Seismographic Network, https://www.iris.edu/hq/programs/gsn), or wildfires and floods that often can be globally mapped from space due to their size and characteristics [23,24], landslide activity is not monitored at the global scale. Landslides are frequently triggered by other natural hazards like earthquakes, floods, or hurricanes, making the reporting of individual landslides difficult and their effects often indistinguishable from the triggering hazard [2]. Furthermore, landslides are usually small, widespread, and frequent, making it impossible with current technologies and funding to monitor and map landslides globally every day. A single storm can generate tens of thousands of landslides [7,25]. Using the GLC methodology, recording a single landslide event takes 20 minutes on average, which comprises of the time it takes to approximate the location of the landslide from the news report and input all relevant details from the report. The addition of all 11,500 landslides to the GLC has taken the GLC team a cumulative 1.6 years of 8-hour workdays to report.

Another challenge is identifying landslides in areas with limited imagery or data, limited newspaper reporting, and/or in remote areas, creating spatial and temporal bias to the landslide inventory data [6–9]. The GLC is affected by reporting biases that impact the spatial distribution of data in addition to the challenges listed for all landslide inventories [14]. These problems include reliance on reports written in English, improved reporting closer to populated areas or transportation networks, increased likelihood of reporting when human life and property is involved, whether reporting networks exist, and bias from political or economic differences among regions [12,14]. During validation of the Landslide Hazard Assessment for Situational Awareness (LHASA) model, Kirschbaum et al. (2018) found that many areas of modeled high landslide potential had few reports of past landslide events. The authors concluded that a spatially consistent landslide inventory is “of foremost importance” to advance the LHASA model [16]. Citizen science was used in this project to extend data collection beyond the limitations of current methods.

### Enhancing the GLC with citizen science

Citizen science—or “crowd science”, “community science”, among other terms—is an increasingly popular methodology that is enabling the public to participate in the massive collection or analysis of data and solutions to scientific problems [26,27]. In the last two decades the number of publications involving citizen science projects increased by a factor of one hundred, attributed to the increasing use of citizen science data by professional researchers, the availability of technical tools, and the emphasis on better science outreach and communication [28,29]. The wide use of and access to the Internet, the Global Positioning System (GPS), and geospatial/mapping programs like Google MyMaps (https://www.google.com/mymaps) or Esri ArcGIS (https://www.esri.com/) have enabled the public to crowdsource geographic information online for purposes including citizen science [30]. To obtain data of sufficient quantity and quality, citizen science projects must raise the public’s interest and provide standard methods for data documentation and access. However, there are many examples of successful citizen science projects that have addressed these concerns with targeted outreach, proper project management, improving attitudes towards citizen science, and implementing standard training protocols and data quality checks [31,32]. Overall, the citizen scientist public gives scientists an opportunity to explore vast amounts of data and analysis tied to location and time-based information to advance research. Results from eBird (https://ebird.org/), a bird-sighting citizen science project, have been robust enough to visualize country-wide patterns in bird species distribution with more data than could be collected by traditional research [33].

Like eBird’s influence on ornithology research, citizen scientists can help collect greater quantities of landslide data while specifically addressing some of the limitations pervasive in existing landslide cataloging efforts and the GLC. First, the sheer number of potential citizen scientists can capture a much greater quantity of data contributed, which can enable a much faster and less resource-intense mapping of large numbers of landslides. More people to collect landslides minimizes the burden on the NASA landslide team, allowing for a more up-to-date global catalog and for rapid response during major triggering events. *In situ* reports made by citizen scientists can add information about locations that cannot be visited by researchers to strengthen the detail and accuracy of submitted data and helping to remove event uncertainty, such as recording the state of the weather, approximate date, the surrounding environment, the movement and possible cause of the landslide, and the photographing of the slide [34]. Finally, citizen scientists may also capture reports from non-English speaking media, helping to address some regional biases in the GLC.

Landslide citizen science has already been applied across a variety of regions and methods. Table 1 lists all landslide citizen science projects that could be identified through internet searches. The scope and duration of these efforts vary widely and exemplify the diversity of citizen science approaches for landslide studies. The projects outlined in Table 1 are generally led by scientists and use simple submission systems. Fourteen of the 20 projects identified were government-led, university groups ran five projects, and a non-governmental organization (NGO) led one project. The top-down approach of landslide citizen science suggests that institutions are important for organizing (and funding) a project at the start and constructing the research question(s) and infrastructure. By doing so, institutions with professional knowledge of landslide hazard and risk assessment can define what types of data are needed to address their scientific questions and create a platform suitable for their target community. Projects vary in scale but they tend to ask citizen scientists for the submission of landslide event information through a document or via email, because reducing the level of professional expertise needed for data submission allows for the widest amount of participation [27,35].

**Table 1:** List of all known^a^ landslide citizen science projects.

Project Name	Institution Name(s)	Scope and Duration	Citizen Science Activity	Study/ Website
Report a Landslide to AGS^b^	Arkansas Geological Survey (AGS)	Government-led Active to present State level	Report landslides to an online Google form, and email photos.	http://www.geology.ar.gov/geohazards/landslides.htm
GeoSocial^b^	British Geological Survey (BGS)	Government-led Active to present National level	The web application filters for geoscience-related posts on social media sites and displays them on a map.	http://www.bgs.ac.uk/citizenScience/geosocial/home.html
Report a Landslide^b^	British Geological Survey (BGS)	Government-led Active to present National level	Report landslides to a form on the webpage, to find new landslides for the national landslide inventory.	[36] http://www.bgs.ac.uk/landslides/report.html
Crowdmap/ GeoExposures^b^	British Geological Survey (BGS)	Government-led Active to present National level	Report temporary geological exposures or geological hazards like landslides, flooding, or rock exposures to an open-source Ushahidi web application and data portal.	[37] http://www.bgs.ac.uk/citizenScience/crowdmap.html
Satark Landslide-warning Project^b^	Centre for Citizen Science (CCS) Pune	NGO-led Active 2013 to present State level	A group of 10 people collected soil samples, readings of wind velocity, and interview locals to report landslide hotspots to a local inventory.	https://satarkindia.wordpress.com/
SIMMA—Sistema de Información de Movimientos en Masa	Colombian Geological Survey	Government-led Active 2015 to present National level	Upload landslide information, location, and photos that occur in Colombia. Data stored in same portal for consulting about regional landslide hazard.	http://simma.sgc.gov.co/
Report a Landslide	Geoscience Australia (GA)	Government-led Active to 2018 National level	Report landslides by emailing a description and photos for the national landslide database.	No active link
Landslide Monitoring App (LaMA)^b^	Hacettepe University	University-led Active October 2018 to present National level	Report landslides to a mobile application. Data will go to an inventory for improving regional landslide susceptibility mapping and characterization.	[38] https://geocitsci.com/
Map the Neighborhood in Uttarakhand (MANU)	HFB Garhwal University in Alaknanda; Kumaun University; Wadia Institute of Himalayan Geology	University-led Active June 2013 to final report on April 2015 State level	Faculty members and 200 students conducted field reporting of landslides, erosion, and damages and reported the data to the Bhuvan geo-portal after the June 2013 flooding from heavy rain.	[39] http://bhuvan.nrsc.gov.in/bhuvan_links.php
Landslide Environmental Virtual Observatories (EVO)^b^	Imperial College, Tribhuvan University, and partners	University-led Funded Sept 2016 to March 2021 Basin level	Use sensor technologies to monitor and collect data in the Karnali Basin, western Nepal.	[40] http://paramo.cc.ic.ac.uk/landslide/
Report a Landslide^b^	Kentucky Geological Survey (KGS)	Government-led Active to present State level	Report landslides to an online form or print out the form and mail it.	http://www.uky.edu/KGS/landslide/
Landslide Inventory Questionnaire^b^	Maine Geological Survey	Government-led Active to present State level	Report landslides to a form on the webpage to help update the state landslide inventory.	http://www.maine.gov/dacf/mgs/hazards/landslides/index.shtml
Landslide Reporter^b^	National Aeronautics and Space Administration (NASA)	Government-led Active March 22, 2018 to present Global level	Report landslides from observations or online to a form on an Esri web application. Data goes to update the Cooperative Open Online Landslide Repository (COOLR), a global landslide inventory of inventories on the web application Landslide Viewer.	https://landslides.nasa.gov
Induced Hazards Team: NASA response to 2015 Nepal earthquakes	National Aeronautics and Space Administration (NASA) and University of Arizona	Government-led Active April 25, 2015 to May 20, 2015 National level	Around 50 scientist volunteers from universities and government agencies in eight countries mapped landslides from satellite imagery as part of a NASA-led disaster response to the 7.8-magnitude Nepal earthquake and its aftershocks on April 25, 2015.	[1] https://www.nasa.gov/feature/goddard/nasa-led-volunteers-map-landslides-by-nepal-quakes
SERVIR-Mekong Myanmar Mapathon	National Aeronautics and Space Administration (NASA) and SERVIR-Mekong	Government-led Active July 2018-August 2018 National level	Two all-day mapathons were held to locate landslides from Google Earth imagery in Myanmar. The landslides were quality checked and added to a landslide inventory hosted in COOLR.	https://landslides.nasa.gov/viewer
歷史影像平台^b^	Taiwan Soil and Water Conservation Bureau (SWCB)	Government-led Active September 2015 to present National level	Upload photographs of landslides with description and location information to a website and data portal.	[41] https://photo.swcb.gov.tw/Repository/Database
Landslide Information System (LIS)^b^	The Hong Kong University of Science and Technology (HKUST)	University-led Active 2016 to present City level	Report landslides to a mobile application. Data goes into a landslide inventory. The next phase of research will use sensors at high-risk sites.	[42]
Did You See It?	U.S. Geological Survey (USGS)	Government-led Active July 2012 to 2015 National level	Report landslides you see to a form on the webpage to raise awareness and contribute to a landslide inventory in the future.	[43]
Generating landslide inventory by participatory mapping: an example in Purwosari Area, Yogyakarta, Java	Universitas Gadjah Mada and Kyushu University	University-led Active 21 days Area level	Three teams of two people mapped landslides for the Purwosari area, Yogyakarta, Java, to verify landslides reported to the Indonesian Disaster Data and Information Database (DIBI) between 1978 and 2011.	[44]
Landslides Inventory GeoForm^b^	Vermont Geological Survey (VGS)	Government-led Active to present State level	Report landslides to an online form on an Esri web application and data portal.	http://dec.vermont.gov/geological-survey/hazards/landslides
Report a Landslide^b^	Wyoming State Geological Survey (WSGS)	Government-led Active to present State level	Report new landslides using a Google form or a printable form to mail, to help update the state landslide inventory.	http://www.wsgs.wyo.gov/hazards/report-landslide

^a^The list was compiled by performing an exhaustive search for relevant pages and publications from combinations of the keywords “landslide”, “mudslide”, “debris flow”, “citizen science”, “crowd science”, “crowdsourcing”, “report a landslide”, “volunteer mapping”, and “participatory mapping” through Google (https://www.google.com/), Google Scholar (https://scholar.google.com), Web of Science (http://www.webofknowledge.com/), and Twitter (https://twitter.com). Searches for a landslide citizen science page or “Report a Landslide” pages were also conducted on the websites of the geological surveys of all 50 states of the United States. Projects were considered relevant to this study if the public was contributing to a landslide inventory for the project to be used for scientific research.

^b^The project is currently active.

In this paper, we present the methods and initial findings of the new Cooperative Open Online Landslide Repository (COOLR) project with a focus on its component, the citizen science platform Landslide Reporter. There are three main objectives to this manuscript: introduce the COOLR project and its methods as a tool for building and sharing a global landslide inventory using citizen science; present the preliminary results of the Landslide Reporter project as a proof-of-concept; and discuss future improvements to the project to advance landslide data gathering with citizen science.

## Materials and methods

### The COOLR project

**Fig 2:**
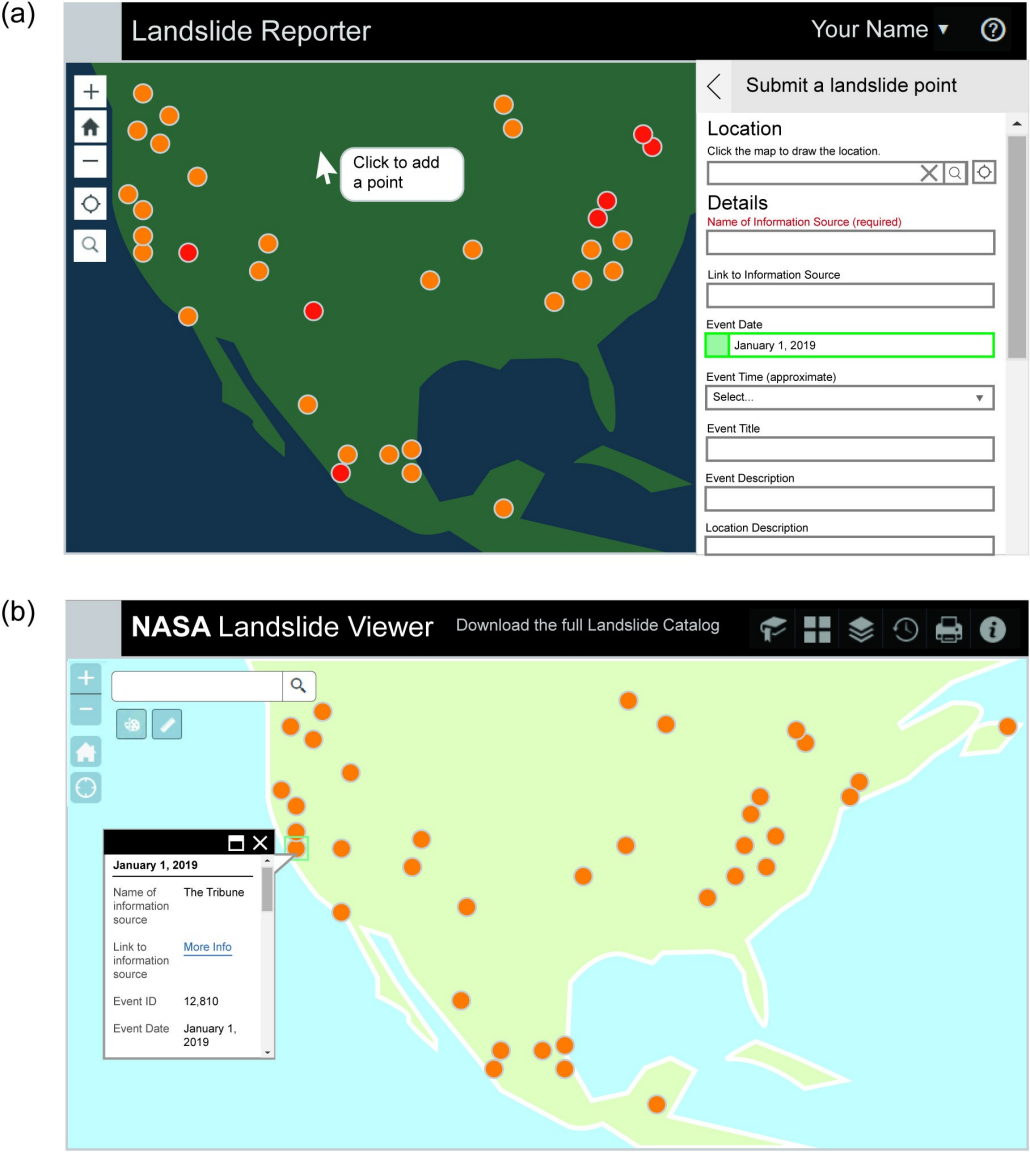
Web application components of the Cooperative Open Online Landslide Repository (COOLR). The figures outline the general design of the web applications for Landslide Reporter and Landslide Viewer: (a) illustration of Landslide Reporter showing the form to report a new landslide, and (b) illustration of Landslide Viewer with the Landslide Points layer visible.

The Cooperative Open Online Landslide Repository (COOLR) was launched on March 22, 2018 and includes Landslide Reporter (https://landslides.nasa.gov/reporter) and Landslide Viewer (https://landslides.nasa.gov/viewer) applications. Collectively, COOLR and its components are designed for the reporting and sharing of worldwide landslide data. COOLR is an open repository for landslide events, including data from NASA’s GLC, citizen scientist-contributed reports, and other publicly available or shared landslide inventories. Landslide Reporter is the citizen science web application used to report landslide events to COOLR, which are stored and visualized in a separate web application, Landslide Viewer, along with other landslide data and remote sensing products. Fig 2 shows examples of the Landslide Reporter (Fig 2A) and Landslide Viewer (Fig 2B) application interfaces. Landslide Reporter is the first and only landslide citizen science project that is global in scope.

### Data storage and web application design

COOLR data are stored at the NASA Center for Climate Simulation (NCCS), and served through in-house ArcGIS servers, at https://maps.nccs.nasa.gov/server/rest/services/ in the folder “global_landslide_catalog”. The landslide repository can be downloaded from Landslide Viewer, which is automatically updated two times a week. Alternatively, the data are also available on NASA’s Open Data Portal (https://data.nasa.gov) under “Global Landslide Catalog,” which is updated less frequently. Landslide Viewer has the most updated version of COOLR and the GLC.

Landslide Viewer was built using the Foldable Theme on Esri Web AppBuilder. Landslide Reporter was customized from the Esri Crowdsource Reporter application (https://github.com/Esri/crowdsource-reporter). COOLR data are available for download in a file geodatabase (.gdb) format, comma-separated values (.csv) format, and shapefile (.shp) format. An additional data reference file is included to provide attribution for inventories that have been directly added to the catalog and provided from outside groups.

### Repository design

The GLC served as the model for structuring the data fields during the creation of the repository. A modified version of the GLC data fields presented in Kirschbaum et al. (2015) is used as the input fields for COOLR. The fields included in COOLR are presented in Table 2. More information about the data fields can be found in Kirschbaum et al. (2010, 2015). Between the publication of Kirschbaum et al. (2015) and the launch of the COOLR project, the “very large” landslide size category was separated into “very large” and “catastrophic” landslides. At the launch of the COOLR project, a new definition to quantify the landslide size categories was appended to each of the size categories to standardize the category and reduce uncertainty when a value for the volume is given. The size category quantifications were informed by observing the reported volumes or reported number of trucks needed to haul away debris and size classifications in a random sample of past landslide events in the GLC. To establish a minimum volume quantity, we observed that small landslides typically only took a few hours to clear with one standard commercial dump truck, which can hold about 8 to 11 m^3^ of material. To establish a maximum volume, we consulted a list of well-known historical landslides available and observed volumes for catastrophic landslides exceeded one million m^3^ [45,46]. The logarithmic scale of volumes was determined to be the best method to encompass all possible landslide sizes reported to the GLC.

**Table 2:** Summary of fields included in the Cooperative Open Online Landslide Repository (COOLR).

Category	Information on Category
Event ID	Unique ID for each reported landslide event.
Source name	Source of report information, includes news source, field observation (in-person observation), disaster database, personal communication, etc.
Source link	URL link to news report or other online source that has a listing of the report.
Date	Reported year, month, and day that the landslide event occurred. Recorded as M:D:YYYY.
Time	Reported hour and minute of the failure, recorded as HH:MM (24-hour clock, local time).
Event title	Common name given to the landslide event or a descriptive title given by the user submitting the event.
Event description	Includes event information such as dimensions of the landslide, characteristics, impacts, timing or situation resulting in the slope failure and other relevant information.
Location description	Location information such as the nearest geographic location (e.g. village, city, region, landmark) if known.
Location accuracy	This field assigns a qualitative uncertainty in the landslide location based on the estimated circular area over which the landslide realistically occurred, described as a radius from the event coordinates to a given radius (in kilometers). • Location known exactly or within 0.1 km • Location known within 1 km • Location known within 5 km • Location known within 10 km • Location known within 25 km • Location known within 50 km • Location known within 100 km • Location known within 250 km • Location not known
Landslide category	Mass movement types are included if known or specified in the source and includes the most frequently reported types of events^a^: landslide, mudslide, debris flow, rock fall, translational slide, rotational slide, complex, topple, riverbank collapse, lahar, earth flow, snow avalanche, creep, other, or unknown.
Landslide trigger	Includes the most common triggers of landslide events. Only the primary trigger can be specified, other triggers can be added to the event description. These include: rain, downpour, continuous rain, tropical cyclone, earthquake, snowfall/snowmelt, leaking pipe, mining, construction, vibration, freeze/thaw, flooding, dam embankment collapse, volcano, monsoon, no apparent trigger, other, or unknown.
Landslide size	This category is to identify the relative size of the landslide in an attempt to differentiate small landslides occurring in backyards and along roads from larger landslides that have caused catastrophic damage and cover wide areas. The “Size Classification” values are from Kirschbaum et al. (2010), which describe the landslide cataloging methodology. A quantified scale was developed for cases in which volume is reported, and has been used since March 2018^b^. • *Small*: Small landslide affecting one hillslope or small area. 𢗋 Possible damage: Minimal impacts to infrastructure and roads, no fatalities or few fatalities. ○ Volume: <10 cubic meters • *Medium*: Moderately sized landslide that could be either a single event or multiple landslides within an area, and involves a large volume of material. ○ Possible damage: Moderate impact to infrastructure and roads, no fatalities or few fatalities. ○ Volume: 10 to <1000 cubic meters • *Large*: Large landslide or series of landslides that occur in one general area but cover a wide area. ○ Possible damage: Substantial impacts to infrastructure and roads, likely moderate to high number of fatalities, tens to hundreds of people displaced. ○ Volume: 1000 to <100,000 cubic meters • *Very large*: Very large landslide or multiple events that affect an entire region (often encompassing an entire village or larger area) ○ Possible damage: Substantial impacts to infrastructure and roads, high numbers of fatalities, thousands of people may be displaced. ○ Volume: 100,000 to <1 million cubic meters • *Catastrophic*: Catastrophic landslide or multiple events that affect multiple villages, towns, and regions ○ Possible damage: Irreversible damage to infrastructure and roads; catastrophic numbers of fatalities, tens of thousands of people may be displaced. ○ Volume: 1 million cubic meters or greater • *Unknown*
Landslide setting	The surrounding environment on which the landslide occurred. The most common settings are included: above road, below road, above river, above coast, burned area, deforested slope, urban, mine, retaining wall, natural slope, engineered slope, bluff, other, or unknown.
Number of fatalities	Number of reported fatalities as a result of the event.
Number of injuries	Number of reported injuries as a result of the event.
Storm name	Includes the name or number of a tropical cyclone if identified (e.g. Hurricane Sandy, Typhoon Mangkhut, Tropical Depression No. 12).
Photo link	The image address to a photo from the source of the event information, in the form of a URL.
Comments	Comments about the report directed towards end-users about the quality of the report, quality of source information, accuracy of location, etc.
Event import source	Name of the landslide inventory the event is reported in. Field contains detail about where the landslide report came from, whether the GLC, LRC, or imported landslide inventories. • *GLC*: NASA Global Landslide Catalog • *LRC*: Landslide Reporter Catalog; events contributed by citizen scientists
Event import ID	Unique ID from the source where the report is imported from, if source is an imported landslide inventory.
Latitude and longitude	Latitude and longitude of the reported event, in World Geodetic System 1984 (WGS 84).
Country name	Country where the landslide occurred.
Country code	Two-letter ISO alpha-2 country code where the landslide occurred.
Administrative division name	Name of the administrative division of the country where the landslide occurred (e.g. state, province, country, etc.)
Gazetteer closest point	Closest known geographic location (city, town, village, etc.) to the landslide event location.
Gazetteer distance	Distance from the gazetteer closest point to the landslide event location, in kilometers.
Submitted date	Date the landslide event was reported to COOLR.
Last edited date	Date the landslide event was edited in COOLR.

Fields are modified from the Global Landslide Catalog (GLC) [12,14].

^a^Landslide category classifications are modified from Cruden and Varnes (1996) and the USGS (2004) [47,48].

^b^New to this publication, a quantified scale for landslide size category was developed by analyzing the size characteristics of previous landslide events in the GLC.

The most important fields within each report within the GLC and COOLR are the date and location of the landslide, which are important for hazard model validation. Other information such as the trigger and time of the event are helpful to identify a possible triggering event for the landslide [12]. COOLR is separated into two geodatabases, one for landslide event points and one for landslide event polygons.

**Fig 3:**
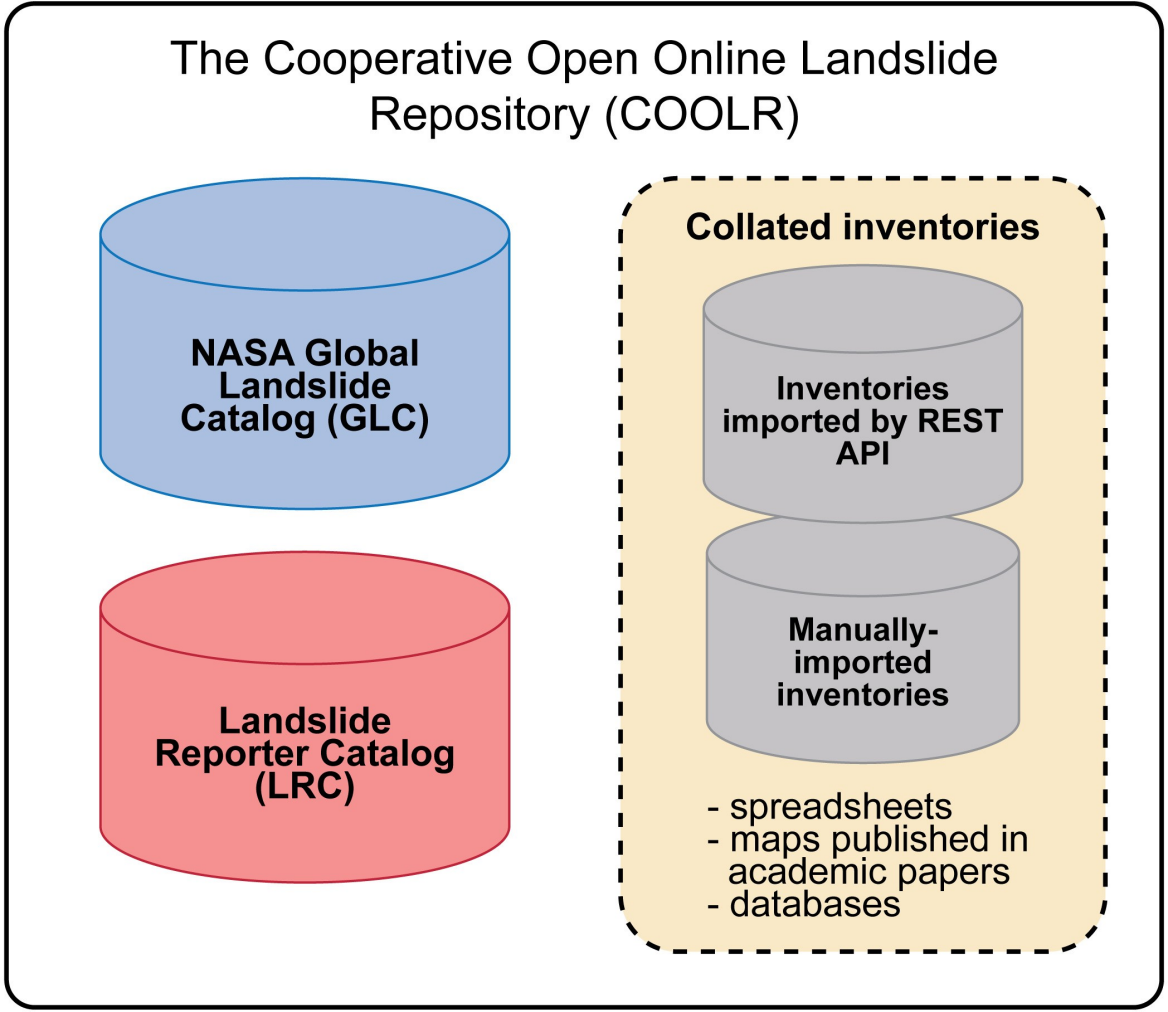
Cooperative Open Online Landslide Repository (COOLR) Components Schematic. The rectangle encompasses the sources of data within COOLR. The three sources are NASA’s Global Landslide Catalog (GLC), citizen scientist contributions through the Landslide Reporter Catalog (LRC), and collated inventories from landslide experts and other citizen science projects.

There are two main sources for data in COOLR—the GLC and citizen science data from Landslide Reporter (Fig 3). The different contributors of the data are specified within the “Event import source” field. GLC data contributed from within NASA are labeled as “GLC”, and Landslide Reporter data contributed by citizen scientists are labeled as “LRC”. After the GLC was incorporated into COOLR, the database was cleaned to remove duplicate landslide events and events where no source was verified. Both the GLC and LRC data are added to the same point or polygon geodatabase. The database is checked prior to a Landslide Reporter submission to ensure that there are not any duplicate reports in the GLC and LRC; however, a single event may be reported separately to the point geodatabase and the polygon geodatabase.

A third component of COOLR involves importing landslide inventories from landslide experts and citizen science projects to create a framework for open landslide data sharing and access (Fig 3). These additional inventories are included through the ArcGIS REST API or through manual import. Landslide inventories hosted on servers external to NCCS are added via their Esri REST API directly to Landslide Viewer. Landslide inventories that must be imported manually and stored with NCCS are fitted to COOLR’s fields (Table 2) and added to COOLR’s geodatabase file. Each manually added landslide inventory is modified into COOLR’s fields on a case-by-case basis and validated with the author of the data prior to entry. A .csv file on the same page as the downloadable repository file lists the citation information for manually added inventories.

### COOLR citizen science and administration process

We target all citizen science audiences to participate (landslide experts and non-experts) that have an education level of middle school and above. Researchers working with landslide data may be motivated to improve the catalog quality for themselves and others since the entire database is easily downloaded in several formats. There are also university professors that have expressed an interest in having their classes participate in this project as part of a lab for the class to teach about using and analyzing geospatial data while contributing to a global cataloging effort. Landslide experts may also be interested in this project because Landslide Reporter may help them keep track of landslides during or after field research. Non-expert citizen scientists have also become interested in participating. They are motivated to contribute in order to learn more about landslides from the introductory materials provided at https://landslides.nasa.gov, address landslide issues within their communities, and/or help scientific research. There are additional ways to incentivize citizen scientists to participate, which we discuss below in “Future improvements to COOLR.”

We seek to attract a diverse public who can fill in gaps in the inventory and alleviate current limitations, and therefore we are not targeting any specific region. We hope to attract citizen scientists, whether living in the area or reading international news reports, who can submit data for regions that currently have a dearth of reports, such as in the Southern Andes, the East African Rift Zone and Turkey and Iran [16]. In contrast, we also welcome landslide reporting for data-rich areas like the United States because we hope regular users will keep the repository up-to-date.

The ancillary goals of using citizen science in COOLR are to educate the public, serve as a teaching tool for educators about landslides, and encourage further scientific exploration. By using Landslide Reporter and Landslide Viewer, citizen scientists can learn how to differentiate the types of mass movements (e.g. mudslide, rock fall, translational slide, etc.) by following the training materials on the website. Additionally, Landslide Viewer provides both the open landslide catalog as well as satellite and model products that can highlight the distribution of landslide hazard and impacts around the world or be downloaded in several different formats.

**Fig 4:**
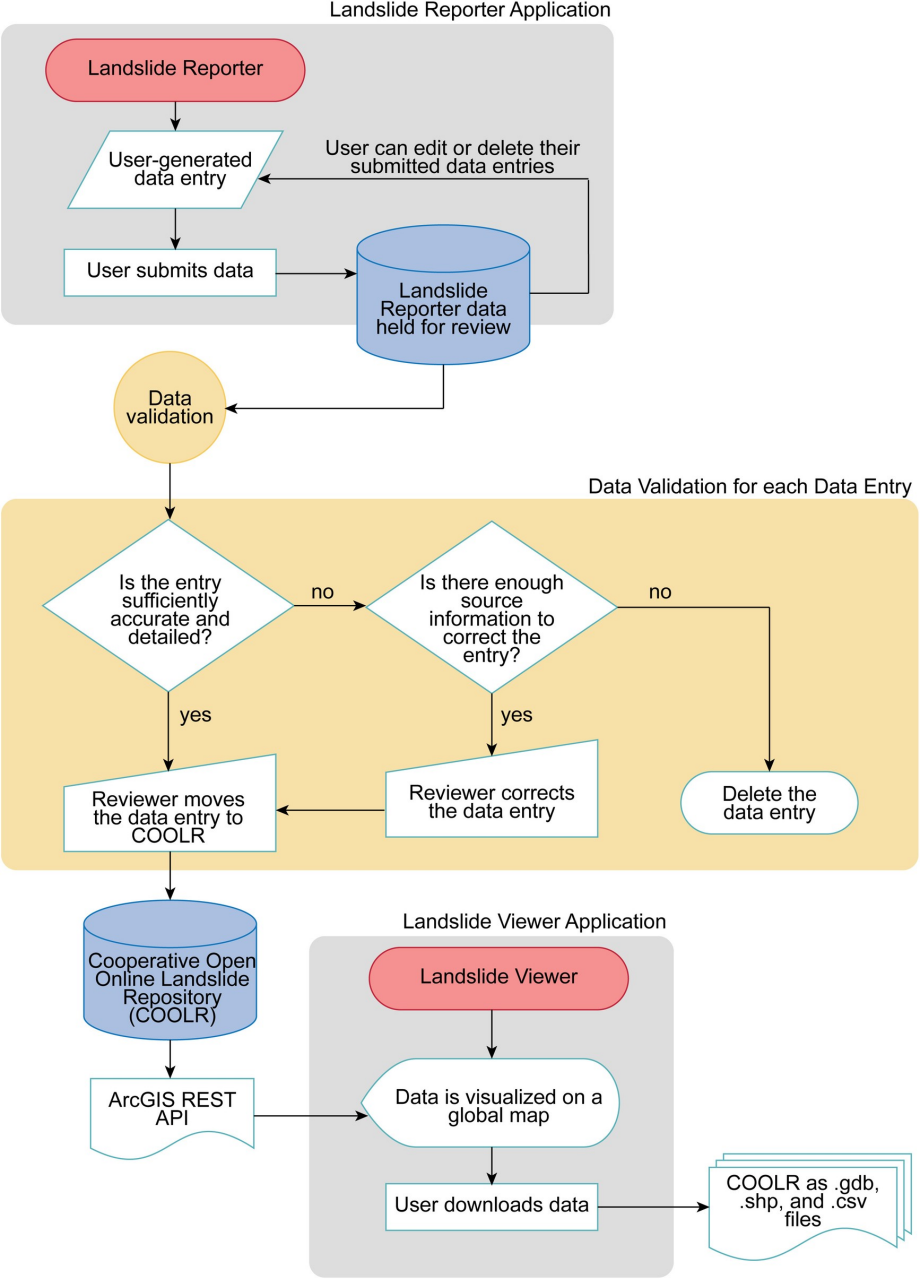
Landslide Reporter submission process. The process is divided into three parts: Landslide Reporter, data validation, and Landslide Viewer. The gray indicates the parts of COOLR’s component web applications that the citizen scientist and the public (the user) can interact with. The yellow indicates the process that a NASA scientist (the reviewer) must conduct in the backend for validating all citizen science data. Landslide Reporter and Landslide Viewer can be accessed independently at any time.

Fig 4 highlights the current submission and validation process for Landslide Reporter. To begin contributing, citizen scientists must log in to access the reporting form on Landslide Reporter. Citizen scientists must accept COOLR’s Landslide System Contribution Policy, Landslide Contributor License Agreement, and the Take Down Policy before using the web application. Upon login with a Google account or Facebook account, each citizen scientist is automatically assigned a unique user ID, a random string of numbers and characters that is associated with their account. The ID is stored and used to find all of a citizen scientist’s previously contributed landslides in the inventory of reports held for review. No personally identifiable information is stored in any part of the system, and the unique user IDs are not included with the downloadable version of the repository on Landslide Viewer.

There are several guides for how to fill in the fields within the application. Training materials are an important asset to help citizen scientists learn how to use the web applications and submit data that are valuable for scientific research [27]. There are five how-to guides currently available on https://landslides.nasa.gov, downloadable as .pdf files:

- **“Primer and Landslide Identification”** teaches readers about why it is important to study landslides and provides information to help fill out the landslide category and landslide size fields.
- **“Add a Landslide Event to COOLR”** explains each field in the report form with guiding images following the contents of Table 2 and how to navigate the web application.
- **“Landslide Identification Training”** is a training protocol for citizen scientists with little to no professional experience. The identification guide gives examples of landslides types (landslide, mudslide, debris flow, and rock fall) and their qualitative sizes (small, medium, large, very large) following the information in Table 2.
- **“Tips to Pinpoint Landslide Location from a News Article”** helps citizen scientists use context clues in a news source to find a reasonable latitude and longitude for the location of a landslide using Google Maps or Google Earth.
- **“Download and Export COOLR”** guide has instructions on how to access the download page on Landslide Viewer, how to cite the data, how to open the .gdb file in ArcMap or QGIS, and convert the file to a .csv.

These guides will be updated as needed. For visual learners, the website also contains videos with an overview for the project, how to navigate the web applications, how to export the data, and how to use the ArcGIS REST API. The website is also a resource to learn more about how the data are used by linking visitors related publications and projects.

Once the citizen scientist completes and submits their report, the data are held for review on the NCCS server. After submission, citizen scientists can still access the report on the server to delete or make edits to the report until the report is validated by the team at NASA. During the validation process, the NASA reviewer will check the entire submission and make any modifications necessary to improve the accuracy or detail of the report (Fig 4). All landslide events in need of validation are stored in an ArcMap database and submissions are held for review. These data are overlaid with a map of previously recorded events in COOLR. Each submitted report is first checked for duplicates (S1 File). If the date of the reported event matches or is very close in date to an event already in COOLR, the descriptions of the events are compared. If it is determined to be a duplicate event, then any new information from the new reported event will be merged into the older event report. Once all the information is extracted, the newer event data will be deleted from the inventory of submissions held for review. For new landslide events, the report is scrutinized for sufficient accuracy and detail (S1 File). This is performed either by comparing the reported information to the source link in the “Source name” and “Source link” fields or by checking the information to the extent possible if the landslide event was an in-person observation. Then, the report will either be added to COOLR or deleted, and updated in Landslide Viewer accordingly.

## Communication and outreach

Landslide Reporter achieved a small following from outreach through social media. At launch in March 2018, the CitizenScience.gov and SciStarter blogs were the first to publicize the COOLR project [49,50]. SciStarter, in turn, publicized their post to the PLOS (Public Library of Science) Blog network’s CitizenSci blog and to Discover’s Citizen Science Salon blog. We also advertised the project on Twitter and Facebook, using hashtags like #citizenscience or #citsci. Subsequent spikes in interest (more than 100 “reblogs” and “likes”) are attributed to the attention received through blog posts written by the American Geophysical Union’s (AGU) The Landslide Blog and the NASA Earth Observatory [51,52]. The NASA Earth Observatory blog post was then shared through Earthsky [53] and their community reblogged this at least 50 more times.

Our existing communication strategy is to use Twitter and Facebook to share Landslide Reporter with the public, send updates about citizen scientist-submitted data, link to educational and relevant articles, and highlight project achievements. We also include our project on open citizen science project databases to help increase interest of and drive traffic towards our project. Currently, Landslide Reporter is part of the Federal Crowdsourcing and Citizen Science Catalog (https://www.citizenscience.gov/catalog/), the SciStarter Project Finder (https://scistarter.com/finder), and the Science by Bike project database (http://sciencebybike.com/explore-projects/). Through these methods, we have attracted the attention of and submissions from landslide professionals, academics, students, and the public, evidence that we are reaching the target audience of both landslide experts and non-experts. Commercial, academic, community volunteer, and government-led landslide groups have expressed interest in our project, and four of these groups have submitted landslide inventories now included in COOLR. One such group, the SERVIR-Mekong team (https://servir.adpc.net/) based in Huntsville, AL, conducted a landslide mapathon to fill in the gaps in Myanmar data in the GLC. The team collected over 1000 points from Google Earth imagery on locations of landslides in the region, transformed the information into the COOLR format, and provided the data for our team to manually include in the repository (Table 1).

We led a successful media blitz in August 2018 to engage more citizen scientists to submit to the project. A Tumblr post and social media stories on the main NASA Snapchat, Facebook, and Instagram shared the importance of studying landslides, connecting this work with other NASA missions such as the Global Precipitation Measurement (GPM) mission. The project linked back to the Landslides @ NASA website to encourage interested viewers to become citizen scientists. The event ultimately brought ~5700 likes and ~1600 shares across Twitter and Snapchat, ~1100 notes (likes and shares) on Tumblr, and 80,000 views on the Snapchat story. These views translated into ~7,400 page views on landslides.nasa.gov and almost 100 new followers across our project’s social media platforms. As a result, there were ~30 new landslide reports made to Landslide Reporter in the first 24 hours of the social media campaign. On the day of the campaign, an extreme rainfall even triggering widespread flooding and landslides occurred in Kerala, India [54]. Landslide Reporter received several reports of this event, highlighting the motivation of citizen scientists to contribute to disaster response.

### Analysis for proof-of-concept

In the results section, we present the preliminary results on landslide events in the LRC from the end of March 2018 (project launch) to the end of November 2018 as a proof-of-concept. Though this is a newly launched system with limited time for data collection, we evaluate the current entries to determine if the citizen scientist-contributed landslide data is of scientific quality and if the data provided can help fill in areas that have a dearth of data. We use metrics of temporal distribution, spatial distribution (distances between events, density of events), the count of source information language and type, location accuracy, and associated landslide susceptibility from Kirschbaum and Stanley (2017) to analyze the data.

The analysis was carried out using the Esri ArcMap and Microsoft Excel. The data analyzed was downloaded from the public repository on Landslide Viewer with the exception of information about the number of citizen scientists. The number of citizen scientists were counted by counting the number of unique user IDs stored in the private version of the repository on the server. Analysis in ArcMap was performed using primarily the Spatial Analyst Package.

## Results and discussion

Over the 13-month study period, citizen scientists have contributed 162 landslide events to COOLR using Landslide Reporter. These events have all been validated and added to COOLR. Additional reports were submitted, but due to incomplete information, duplicates, or the submission of an inventory as one event, they were not added to the repository. Forty-nine participants submitted the 162 landslide events. A bulk of reports were submitted by one “super user” citizen scientist, which corroborates the importance of these individuals within citizen science communities [55,56]. Most other citizen scientists contributed one or two reports. The reported landslides are distributed around the world, suggesting the potential reach this type of platform can have given more time and increased discoverability by citizen science networks. In the following analyses, the new Landslide Reporter Catalog (LRC) data were compared to the NASA Global Landslide Catalog (GLC), containing 11,666 landslide events.

### Filling in gaps in the repository

**Fig 5:**
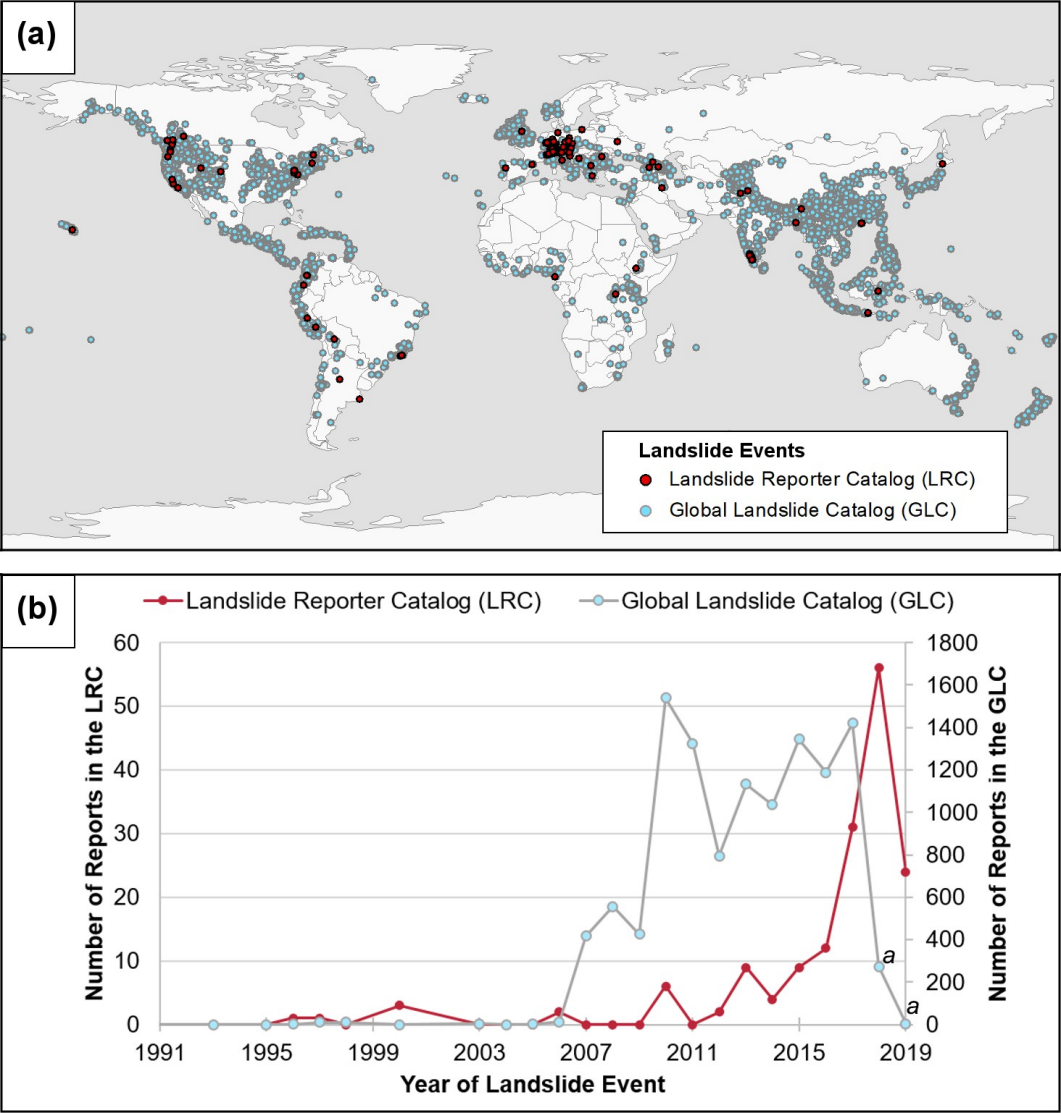
Distribution of landslide events by their location and time. (a) The map shows the spatial distribution of Landslide Reporter Catalog (LRC) events in red and Global Landslide Catalog (GLC) events in light blue. (b) The graph shows the temporal distribution of landslide events in the LRC (in red) in comparison with events in the GLC (in light blue). ^a^The number of landslides in the GLC for 2018 and 2019 is low because we have not yet updated the catalog for this year.

We evaluate the spatial and temporal distribution of the 162 reports we received since March 2018, to determine whether our data are filling in gaps that have been observed in the Global Landslide Catalog. The reported LRC landslides are from 37 countries on five continents (Fig 5A). A time series of these submissions are graphed in Fig 5(B). A landslide that took place in 1897 was excluded from the x-axis so the other yearly data can be seen clearly, and a second landslide was excluded for having no date. Predictably, citizen scientists submitted the greatest number of landslides in 2018 and second-greatest number in 2017. The number submitted in 2019 is steadily rising as the year progresses. The reported landslide events that occurred in previous years indicate that citizen scientists may also seek to fill in gaps in historical records. For example, three citizen scientists submitted information about the Mud Creek Landslide that occurred in Big Sur, California, USA, in May 2017. Though we had to delete the duplicate submissions because they were already recorded in the GLC in COOLR, we moved new details about the slide to the entry. One submission helped correct the location of the slide. These initial findings provide evidence for how citizen scientists are able to enrich the existing repository in space and time.

**Fig 6:**
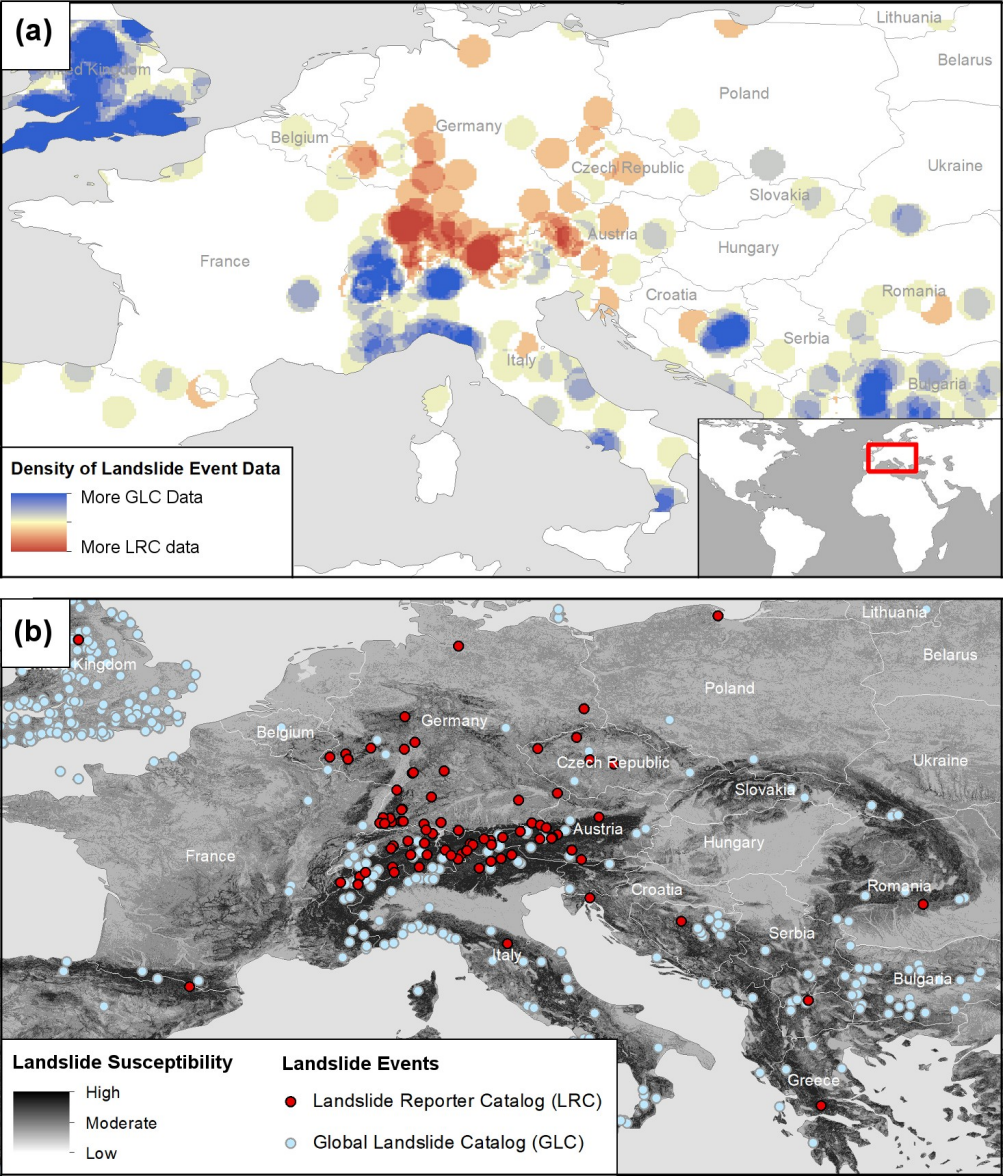
Density and susceptibility of landslide events in Europe. (a) The map shows the spatial density and location of landslide events in Europe for the GLC and LRC. Areas that are darker blue are denser with GLC landslide events. Areas in darker red are dense with LRC landslide events. (b) The map shows LRC events (in red), and GLC events (in light blue) overlaid with the Kirschbaum et al. (2016) [57] landslide susceptibility map.

There is a large number of landslide points submitted to the LRC from Germany and Switzerland, mostly by a single super user. Despite the disproportionality, this fills a gap in the landslide repository within this area. We quantified the spatial density of points from the LRC and the GLC across Europe and subtracted the layers to see where each dataset had a more comprehensive distribution. The resulting map in Fig 6(A) exhibits blue areas where GLC data are present and red areas where LRC data are more prevalent. If the reports are compared with a global susceptibility map provided by Kirschbaum et al. (2016) [57] in Fig 6(B), the LRC reports are superimposed on the Alps and the hilly regions of Germany where landslide susceptibility is high but GLC reports are missing. Both maps visually demonstrate how citizen scientists can help fill in spatial gaps that exist in the GLC.

**Fig 7:**
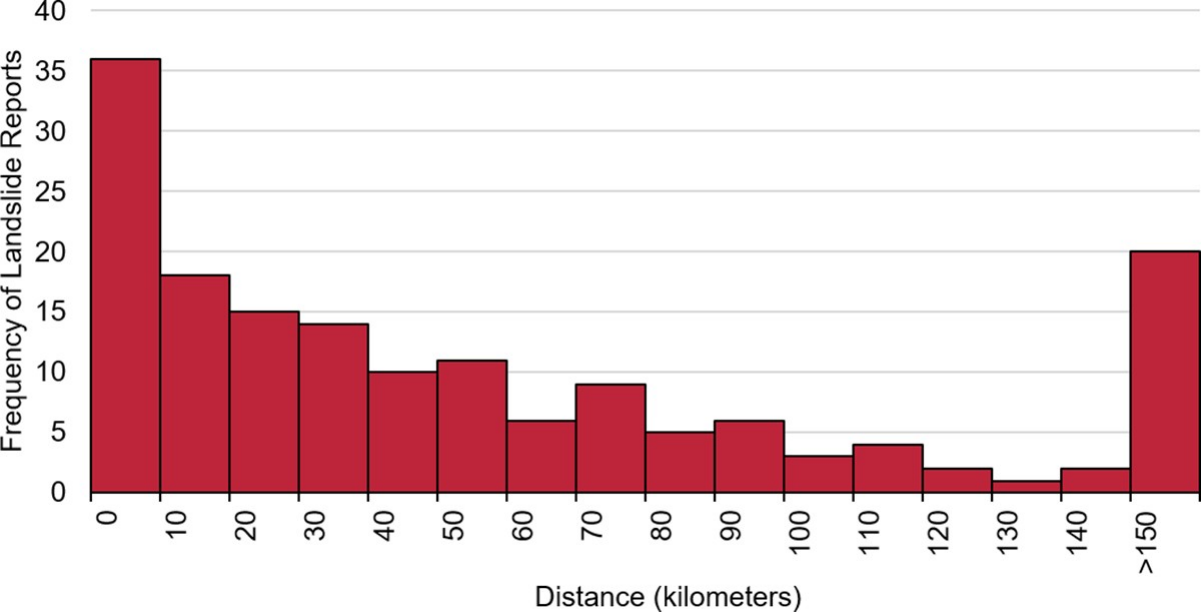
Distance to the nearest GLC landslide event. The histogram illustrates the distance of each LRC landslide event to the nearest GLC event in the repository in bins of 10 km. The last bin contains all distances greater than 150 km to the nearest GLC event.

To further analyze how gaps may be filled on a global scale, we measured the distance of each LRC landslide event to the nearest GLC landslide event. Fig 7 depicts a histogram of all the distances calculated. The greatest number of landslides submitted by citizen scientists occurred within 10 km of any landslide event compiled by our team at NASA. The GLC is robust enough to begin to characterize global patterns in where and when landslides occur, so many LRC events will be located near GLC event data in highly susceptible areas [14]. However, there is a large proportion of events in the LRC that do not neighbour any GLC event, with 20 events extending further than 150 km from the nearest GLC event. The farthest LRC landslide event reported was a cliff collapse in Santa Clara del Mar near Buenos Aires, Argentina, on January 2018, located 1215 km away from any GLC event. The results further suggest that this citizen science method helps to identify new landslide reports in landslide-susceptible areas not currently covered by the GLC.

**Fig 8:**
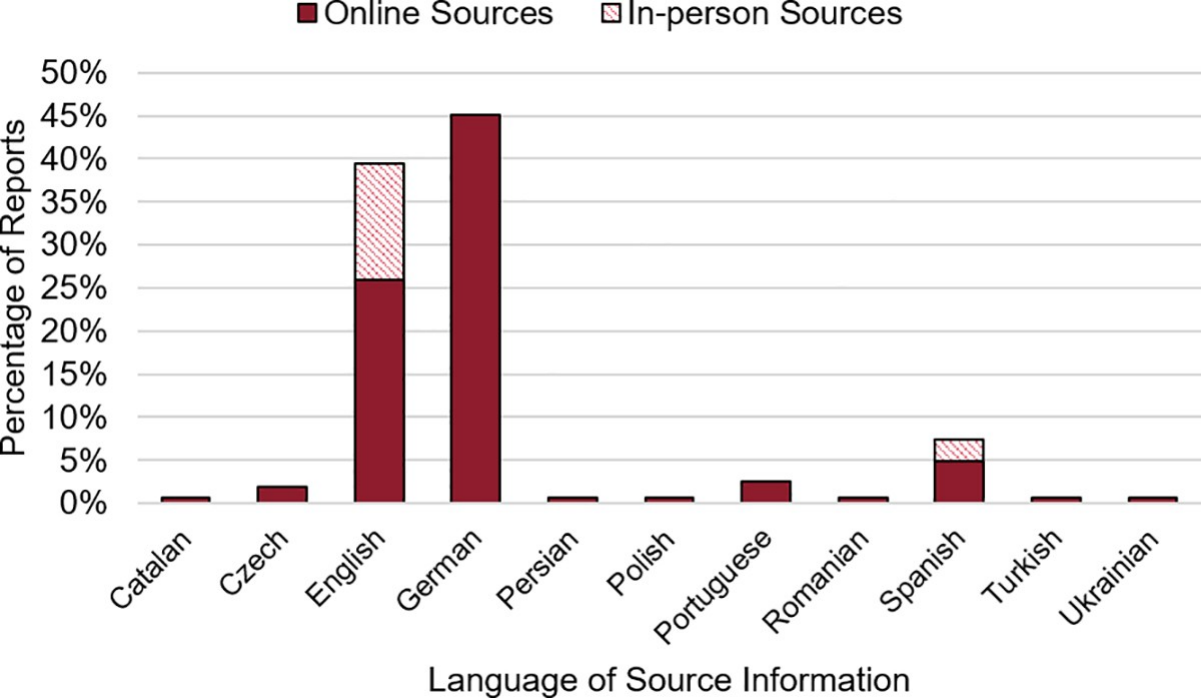
Landslide event sources by language and type of source. The event source specified in the “source name” and “source link” fields for each citizen scientist-contributed report are categorized by language and by the type of source, an in-person observation or an online source.

Lastly, we examined how the challenges of language bias and reporter choice bias may be addressed by citizen scientists. Fig 8 illustrates that more than 60% of reported events in the LRC come from non-English sources. Citizen scientists were able to submit or translate news articles and database entries from Catalan, Czech, German, Persian, Polish, Portuguese, Romanian, Spanish, Turkish, and Ukrainian sources and enter the information into our English web application. Some of the reports are in-person observations, listed with a person’s name or a permutation of “in-person observation” as recommended in our how-to guide. Some of the eyewitness accounts were from landslide experts, submitted with expert details about the slide and sometimes a link to a publication. The initial findings are promising for demonstrating how the known biases in the GLC could be reduced with the help of landslide expert and non-expert citizen scientists who possess language capabilities and/or *in situ* observations to make reports in landslide-prone locations previously overlooked by the GLC methodology.

### Spatial characteristics of submitted landslides

**Fig 9:**
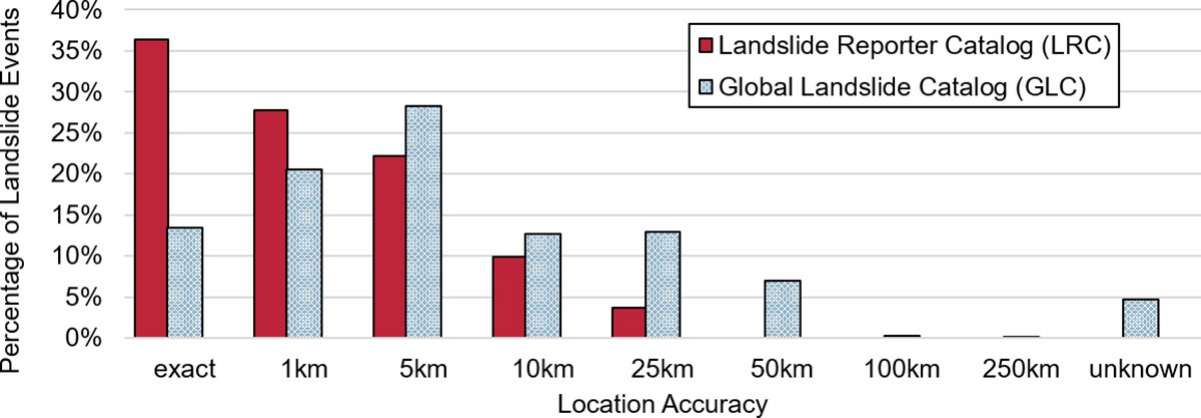
Number of landslide events by location accuracy. The number of landslide events by their “location accuracy” field in the LRC (in red) and the GLC (in blue).

In addition to filling in regional or temporal gaps in COOLR, we want to know if citizen scientists are contributing spatially accurate and viable data for use with landslide maps and models. Fig 9 presents the location accuracy of landslide events in the GLC and in the LRC, a quantitative measure of the citizen scientist’s uncertainty in the latitude and longitude of an event collected at the time of submission to Landslide Reporter (Table 2). The greatest number of reports in the LRC had an “exact” location accuracy, while the greatest proportion of reports in the GLC had a 5 km location accuracy. These initial results suggest that citizen scientists ascertain they can accurately pinpoint landslide locations, an indication that the quality of the reporting in the LRC may enhance the COOLR database. During the data validation process, we noticed that some citizen scientists specified much larger location accuracy values in their reports (25 km and 50 km) but that we could verify these locations from the source article and correct them to a value within 5 km. In the future, new application features such as using built-in measurement tools and changes to the how-to guides may help to improve the characterization of report location accuracy. Nevertheless, validated results indicate that citizen scientists are submitting quantifiably accurate data to the LRC, and the improved spatial accuracy with respect to the GLC could help scientists discern the environmental factors affecting landslides for their scientific models.

**Fig 10:**
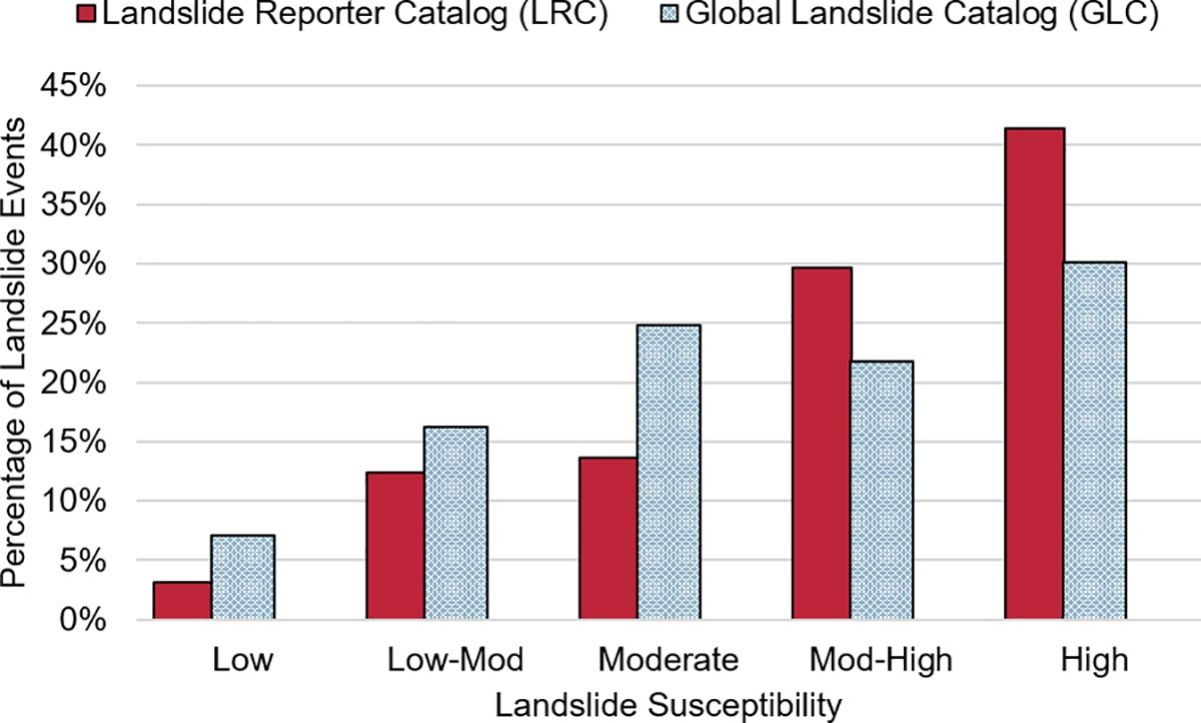
Modeled landslide susceptibility associated with reported events. The landslide susceptibility value from the map produced by Stanley and Kirschbaum [15,57] associated with each LRC (in red) and GLC point (in blue). The events are located on areas of low landslide susceptibility to high landslide susceptibility.

Lastly, we tested how the new LRC data might help improve landslide hazard modeling by superimposing the LRC and GLC data with the landslide susceptibility map produced by Kirschbaum et al. (2016) [15,57]. Data from COOLR will be used to validate the susceptibility map, the LHASA model, and other products [15], so it is valuable to see whether landslides submitted are occurring in predicted landslide-prone areas. In Fig 10, the greatest proportion of LRC events are located in areas with high landslide susceptibility, while the smallest proportion of events are centered in areas of low landslide susceptibility. The results confirm the landslide susceptibility distribution of citizen scientist-contributed events is comparable to that of the GLC, demonstrating that the LRC may help to improve future versions of the landslide susceptibility map, LHASA model, and other modeling efforts within the community.

Despite initial indications that the LRC can help to address gaps in the GLC repository, it is too early in the project to conclude that the citizen science contributions have reduced spatial biases in the repository, and in some cases may have added additional bias due to the citizen scientist’s geographic location (e.g. Germany) and proximity to populated areas. Further, the number of landslide reports in this sample are too small to draw conclusions about other spatial biases like political or economic differences among regions or draw patterns of landslide hotspots. However, the number of in-person observations and reports from non-English sources, the appearance of citizen scientist-submitted reports far from any GLC landslide events, and the density of LRC points within areas not covered by the GLC (Fig 6) suggest that Landslide Reporter may be an important and effective tool to enhance and improve global landslide catalogs as the citizen science database grows over time.

### Future improvements to COOLR

While early results of COOLR—including Landslide Reporter and Landslide Viewer—are promising, many improvements could increase the success of this system and fulfill the project goal of serving as an open repository for global landslide data sharing. First, the COOLR project has begun including other landslide inventories, so a procedure for systematically adding these inventories into COOLR must be created. Inclusion through the ArcGIS REST API is straightforward because the inventory will be hosted elsewhere and then referenced as a new layer on Landslide Viewer. However, an improved manual import strategy is needed to include other landslide inventories directly into COOLR. The landslide report fields prioritized are heavily dependent on the research goals of an institution and may not include some of the key information the GLC and LRC requires, such as a date of the landslide event. Data fields not prioritized by COOLR may end up as free text in the description field. We also foresee that the process of integrating inventories will bring new challenges of spatial bias, temporal bias, and duplicate landslide events. Future work should follow best practices from recent research that analyzes other integrated landslide inventories created from heterogeneous datasets [11,58,59].

To appeal to broader and more diverse audiences, particularly in areas where there is a current dearth of landslide data available, the User Interface (UI) of Landslide Reporter can be improved for a better citizen science experience. The current design of the application is accessible in both web and mobile layouts and has a simple submission form; however, Landslide Reporter requires an internet connection to interact with the form. One solution is to develop a standalone application that can be downloaded onto a mobile phone or tablet to be used *in situ* or in areas of limited connectivity to increase use for fieldwork or disaster responses as well as encourage accurate submissions.

Another future improvement is to expand the interface to be accessible in other languages other than English, whether by human translation or through machine translation services like Google Translate (https://translate.google.com/). Machine translations are not perfect, but they provide a low-cost service when volunteer or paid translators are not available. If the application remains in English, the COOLR project website should provide translations of the how-to guides in other languages. At the time of this publication, we are working with volunteer translators through the newly-formed “Sciencing with NASA” NASA Facebook group (https://www.facebook.com/groups/Sciencing/) to translate a two-page introduction to Landslide Reporter. One volunteer has expressed interest in translating more, so we have asked her to translate the “Add a Landslide Event to COOLR” how-to guide so citizen scientists have access to the full instructions in a native language. We will make these materials available on our website to bring larger international audiences to the project.

Although a simple form has been used by many citizen science projects in Table 1, there may be better ways of collecting landslide data. A future iteration, supported by user feedback, could test different interfaces or data collection methods, such as asking yes/no questions to populate each field (e.g. “Did the landslide occur by a road?”), and having the option to submit or upload photos directly (an important element that is not included within the current architecture). Improving accessibility, transparency, and the usability of Landslide Reporter, along with other user experience changes, can reach greater numbers of potential citizen scientists for this project.

Next, the current setup for approving reports prevents citizen scientists from tracking all of their previously contributed landslide events and it does not send feedback on the status of newly contributed reports. Feedback is critical to the success of the project, because it lets citizen scientists know that their submissions are accurate and contributing to science, a significant motivator for participation [60]. A future version of Landslide Reporter should include an engaging User Interface where citizen scientists can see the total number of reports they contribute to COOLR and receive feedback on why their previously contributed reports were accepted, rejected, or modified during the quality control/data validation step in the process. “Gamifying”—or adding in elements of a game—the entire process may also encourage greater participation or more frequent participation from those who like to score points, compete with their peers, or collect prizes while doing citizen science [61]. Citizen scientists could be motivated to submit the greatest number of landslides if they see other users’ total number of approved landslides on a leaderboard. Another potential motivation and feedback method is to reward citizen scientists with tangible prizes like badges or certificates. Such user-facing elements would improve the quality of reports received, further motivate citizen scientists who are driven by a desire to contribute to science, and show appreciation for citizen scientists’ time and effort.

An exciting future avenue in which we hope to move is using Landslide Reporter for disaster response and mapping efforts, leveraging available remote sensing imagery and other sources to dynamically map landslides that can be immediately validated and seen by emergency response teams. Following a disaster, we envision that Landslide Reporter’s polygon submission form could be used to systematically and collectively map landslides triggered by a major earthquake or storm, bringing in disaster-specific remote sensing data. It could also allow participants to validate other citizen scientist submissions so that data approval is timely. One example where this tool could have improved coordination and data sharing and dissemination following a disaster was the 2015 Nepal earthquake in which a 7.8-magnitude earthquake and its aftershocks resulted in widespread landsliding. A citizen science team of over fifty experts from around the world formed an Induced Hazards Team (Table 1) and used high-resolution imagery to map landslides that may affect disaster response and recovery activities [1]. We can also look to the successes of disaster mapping projects like Tomnod (http://www.tomnod.com/) and Humanitarian OpenStreetMap Team (https://www.hotosm.org/) for their best practices. Leveraging the power of citizen scientists, it could be possible to overcome difficulties in post-disaster landslide inventory collection like the mapping of hundreds of thousands of landslides triggered in a single event [62] or inaccuracies in landslide locations of the events in the GLC.

While increasing the functionality and accessibility of the User Interface is vital to expanding the reach and success of COOLR, the discoverability of this system is also fundamental. Therefore, we will develop new strategies to locate and attract potential citizen scientists for COOLR. We will continue to examine the successes of other citizen science data collection projects like eBird, NASA’s GLOBE Observer (https://observer.globe.gov/), and the Community Collaborative Rain, Hail & Snow Network (CoCoRaHS) (https://www.cocorahs.org/) to learn more about how they are reaching out to new citizen scientists. We may also be able to leverage their and other citizen science networks to promote this project. Future outreach should focus on how to involve more community groups and educational groups beyond social media who could potentially benefit from collecting and using the data. High school and university professors are also an important group to target as they could incorporate COOLR into their Earth science course curriculums and labs to have students contribute to the repository as well as teach data analysis techniques. Furthermore, it has been studied that citizen scientists are driven to contribute by both intrinsic (desire to learn, do a hobby, meet people, impart knowledge, and help others) and extrinsic (desire to gain experience for their career) motivations. Thus, additional social research could focus on the specific motivations compelling citizen scientists to contribute to Landslide Reporter [63].

## Conclusions

The COOLR project represents a unique approach to provide openly available landslide inventories to the community as well as encourage citizen science participation to expand and improve landslide data, given that it is the first landslide citizen science project on a global scale. The three main objectives for this manuscript were to (1) introduce the COOLR project and its methods as a tool for building and sharing a global landslide inventory using citizen science; (2) present the preliminary results of the Landslide Reporter project as proof-of-concept; and (3) discuss future improvements to the project to advance landslide data gathering with citizen science. To fulfill the first objective, this paper documented the setup of the Landslide Reporter and Landslide Viewer web applications, the repository design, and the citizen science methods. Project outreach is performed using the widely-used social media sites Facebook and Twitter with the help of news posts, NASA social media accounts, and citizen science project catalogs.

Next, preliminary results from the 162 new landslide events collected by 49 participants in the first thirteen months of operation demonstrated Landslide Reporter’s ability as a successful citizen science project for spatially and temporally diverse data that fills in data gaps and challenges known data biases. The collected events exhibited a wide distribution with events that occurred in 37 countries and from 1897 to 2019. Duplicate events submitted by multiple citizen scientists still helped enrich the repository with new details about an event. A closer look at the density of events that occurred in Europe, submitted mostly by one enthusiastic citizen scientist, revealed that citizen scientist-contributed data is already filling in spatial gaps in the NASA Global Landslide Catalog (GLC) data. It also revealed the important role of citizen science “super users” working in tandem with citizen scientists who submit one or two reports. Furthermore, some of these Landslide Reporter Catalog (LRC) events were located hundreds to thousands of kilometers away from any existing GLC landslide events. Observations from social media and from the source information submitted with landslide events indicated we are reaching target audiences of landslide experts and amateurs around the world who brought news sources and personal observations in 11 languages into COOLR. More than 60% of reported events contributed by citizen scientists came from non-English sources and roughly 15% came from in-person sources, showing promise that language and newspaper reporting biases to the GLC may be reduced with the help of citizen scientists. An analysis of data location accuracy, a metric collected at the time of submission, revealed citizen scientists submitted proportionally more spatially accurate landslides in comparison to the GLC. Lastly, superimposing LRC and GLC events on the landslide susceptibility map produced by Stanley and Kirschbaum [15,57] depicted both catalogs have similar proportions of high, moderate, and low susceptibility events, corroborating the spatial accuracy of the citizen scientist data. This study analyzed the results of the first year since project launch, so it is too early to draw conclusions on how citizen science is handling other biases noted by Kirschbaum et al. [14]. However, these initial results show proof-of-concept for how this platform may help to address data gaps created by collecting reports through the GLC methodology through in-person observations, non-English sources, and the appearance of gap-filling LRC reports far from any GLC landslide event.

The global and open nature of COOLR means that the project could serve as a centralized place for the collection and dissemination of landslide data on local to global scales. We envision that researchers could use Landslide Viewer to locate existing landslide inventories and landslide data for an area of interest. Landslide Viewer has already begun collecting regional and state-wide landslide inventories by referencing their Esri ArcGIS REST API or by including them directly into COOLR. At the time of this publication, four state landslide inventories are referenced and four landslide inventories from academic and community volunteer groups are imported into COOLR, all available on Landslide Viewer. In parallel with Landslide Viewer, Landslide Reporter could be used as a central location for contributing landslide data, which can be especially useful to groups who do not have such a system in place locally for submitting such information.

At this time, the COOLR project’s reach is constrained by the English language, and the web application could use other features to make collecting landslides in Landslide Reporter more engaging considering the limited number of submissions received over the first year. Therefore, the third objective of this manuscript was to discuss ideas to expand and evolve COOLR in directions that will improve the types of data collected, how it is collected, the quality of the submissions, and the number of submissions. Presently, we are bringing in volunteer translators to create a two-page document in other languages describing Landslide Reporter to reduce language barriers to use the application. Future versions of this project should strategize integrating other landslide inventories into the repository that have different fields in their databases. We also foresee improving the user experience by allowing users to access their past submissions, incentivizing citizen scientists through game elements in the application, inviting expert citizen scientists to participate in data validation, and adding in machine or human translation. Finally, we hope to include collective post-disaster mapping in Landslide Reporter, allowing citizen scientists to contribute to post-disaster response, recovery, and mitigation.

Given that COOLR is a research project and subject to the same funding processes as other research grants, the evolution of future improvements to COOLR outlined above are still unknown. Nevertheless, we are encouraged by the preliminary results and feel that citizen science is an optimal path for improving the GLC and opening up more landslide data to the global community. We hope to continue the project and will work with national and international partners and stakeholders to examine the different ways in which COOLR may evolve. We hope COOLR will become a highly useful tool for collecting and sharing landslide data on all geospatial scales and substantially improve access to and accuracy of landslide information that can be used for risk assessment, research, and decision-making to save lives and property.

## Supporting information

S1 File Data validation protocol. The detailed protocol used for checking all citizen science data contributed to the Cooperative Open Online Landslide Repository (COOLR) through the Landslide Reporter application as of February 2019.
(PDF)

## References

[1] Kargel JS, Leonard GJ, Shugar DH, Haritashya UK, Bevington A, Fielding EJ, et al Geomorphic and geologic controls of geohazards induced by Nepal’s 2015 Gorkha earthquake. Science (80-). 2016;351: aac8353-1–aac8353-10. 10.1126/science.aac8353 26676355

[2] Kjekstad O, Highland L. Economic and Social Impacts of Landslides In: Sassa K, Canuti P, editors. Landslides–Disaster Risk Reduction. Berlin, Heidelberg: Springer-Verlag; 2009 pp. 573–587. 10.1007/978-3-540-69970-5

[3] Petley DN. Landslide hazards In: Alcantara-Ayala I, Goudie A, editors. Geomorphological Hazards and Disaster Prevention. Cambridge, UK: Cambridge University Press; 2010 pp. 63–74. 10.1017/CBO9780511807527.006

[4] Cruden DM. A simple definition of a landslide. Bull Int Assoc Eng Geol. 1991;43: 27–29. 10.1007/BF02590167

[5] van Westen CJ, Castellanos E, Kuriakose SL. Spatial data for landslide susceptibility, hazard, and vulnerability assessment: An overview. Eng Geol. 2008;102: 112–131. 10.1016/j.enggeo.2008.03.010

[6] Malamud BD, Turcotte DL, Guzzetti F, Reichenbach P. Landslide inventories and their statistical properties. Earth Surf Process Landforms. 2004;29: 687–711. 10.1002/esp.1064

[7] van Westen CJ, van Asch TWJ, Soeters R. Landslide hazard and risk zonation—Why is it still so difficult? Bull Eng Geol Environ. 2006;65: 167–184. 10.1007/s10064-005-0023-0

[8] Galli M, Ardizzone F, Cardinali M, Guzzetti F, Reichenbach P. Comparing landslide inventory maps. Geomorphology. 2008;94: 268–289. 10.1016/j.geomorph.2006.09.023

[9] Guzzetti F, Mondini AC, Cardinali M, Fiorucci F, Santangelo M, Chang KT. Landslide inventory maps: New tools for an old problem. Earth-Science Rev. Elsevier B.V.; 2012;112: 42–66. 10.1016/j.earscirev.2012.02.001

[10] Ibsen M-L, Brunsden D. The nature, use and problems of historical archives for the temporal occurrence of landslides, with specific reference to the south coast of Britain, Ventnor, Isle of Wight. Geomorphology. 1996;15: 241–258. 10.1016/0169-555X(95)00073-E

[11] Tanyaş H, van Westen CJ, Allstadt KE, Anna Nowicki Jessee M, Görüm T, Jibson R, et al Presentation and Analysis of a Worldwide Database of Earthquake-Induced Landslide Inventories. J Geophys Res Earth Surf. 2017;122: 1991–2015. 10.1002/2017JF004236

[12] Kirschbaum DB, Adler R, Hong Y, Hill S, Lerner-Lam A. A global landslide catalog for hazard applications: Method, results, and limitations. Nat Hazards. 2010;52: 561–575. 10.1007/s11069-009-9401-4

[13] Petley DN. Global patterns of loss of life from landslides. Geology. 2012;40: 927–930. 10.1130/G33217.1

[14] Kirschbaum DB, Stanley T, Zhou Y. Spatial and temporal analysis of a global landslide catalog. Geomorphology. Elsevier B.V.; 2015;249: 4–15. 10.1016/j.geomorph.2015.03.016

[15] Stanley T, Kirschbaum DB. A heuristic approach to global landslide susceptibility mapping. Nat Hazards. Springer Netherlands; 2017;87: 145–164. 10.1007/s11069-017-2757-y PMC8051514 33867675

[16] Kirschbaum DB, Stanley T. Satellite-Based Assessment of Rainfall-Triggered Landslide Hazard for Situational Awareness. Earth’s Futur. 2018;6: 505–523. 10.1002/2017EF000715 PMC6839699 31709272

[17] Guha-Sapir D, Below R, Hoyois P. EM-DAT: International Disaster Database. In: Catholic University of Louvain: Brussels, Belgium [Internet]. 2015 [cited 31 May 2018]. Available: https://www.emdat.be/

[18] Monsieurs E, Jacobs L, Michellier C, Basimike Tchangaboba J, Ganza GB, Kervyn F, et al Landslide inventory for hazard assessment in a data-poor context: a regional-scale approach in a tropical African environment. Landslides. 2018; 1–15. 10.1007/s10346-018-1008-y

[19] Cullen CA, Al-Suhili R, Khanbilvardi R. Guidance index for shallow landslide hazard analysis. Remote Sens. 2016;8: 1–17. 10.3390/rs8100866

[20] Farahmand A, Aghakouchak A. A satellite-based global landslide model. Nat Hazards Earth Syst Sci. 2013;13: 1259–1267. 10.5194/nhess-13-1259-2013

[21] Guzzetti F, Cardinali M, Reichenbach P. The AVI project: A bibliographical and archive inventory of landslides and floods in Italy. Environ Manage. 1994;18: 623–633. 10.1007/BF02400865

[22] Barra A, Monserrat O, Mazzanti P, Esposito C, Crosetto M, Scarascia Mugnozza G. First insights on the potential of Sentinel-1 for landslides detection. Geomatics, Nat Hazards Risk. Taylor & Francis; 2016;7: 1874–1883. 10.1080/19475705.2016.1171258

[23] Wang X, Xie H. A review on applications of remote sensing and geographic information systems (GIS) in water resources and flood risk management. Water (Switzerland). 2018;10: 1–11. 10.3390/w10050608

[24] Mouillot F, Schultz MG, Yue C, Cadule P, Tansey K, Ciais P, et al Ten years of global burned area products from spaceborne remote sensing-A review: Analysis of user needs and recommendations for future developments. Int J Appl Earth Obs Geoinf. Elsevier B.V.; 2014;26: 64–79. 10.1016/j.jag.2013.05.014

[25] Bucknam RC, Coe J a, Chavarría MM, Godt JW, Tarr AC, Bradley L-A, et al Landslides Triggered by Hurricane Mitch in Guatemala—Inventory and Discussion. Open File Rep 01–443. 2001; 38.

[26] Franzoni C, Sauermann H. Crowd science: The organization of scientific research in open collaborative projects. Res Policy. Elsevier B.V.; 2014;43: 1–20. 10.1016/j.respol.2013.07.005

[27] Bonney R, Cooper CB, Dickinson J, Kelling S, Phillips TB, Rosenberg K V., et al Citizen Science: A Developing Tool for Expanding Science Knowledge and Scientific Literacy. Bioscience. 2009;59: 977–984. 10.1525/bio.2009.59.11.9

[28] Silvertown J. A new dawn for citizen science. 2009;24: 467–471. 10.1016/j.tree.2009.03.017 19586682

[29] Follett R, Strezov V. An analysis of citizen science based research: Usage and publication patterns. PLoS One. 2015;10: 1–14. 10.1371/journal.pone.0143687 26600041 PMC4658079

[30] Goodchild MF. Citizens as sensors: The world of volunteered geography. GeoJournal. 2007;69: 211–221. 10.1007/s10708-007-9111-y

[31] Conrad CC, Hilchey KG. A review of citizen science and community-based environmental monitoring: Issues and opportunities. Environ Monit Assess. 2011;176: 273–291. 10.1007/s10661-010-1582-5 20640506

[32] Elwood S, Goodchild MF, Sui DZ. Researching Volunteered Geographic Information: Spatial Data, Geographic Research, and New Social Practice. Ann Assoc Am Geogr. 2012;102: 571–590. 10.1080/00045608.2011.595657

[33] Klemann-junior L, Alejandro M, Vallejos V, Scherer-neto P, Ricardo J. Traditional scientific data vs. uncoordinated citizen science effort: A review of the current status and comparison of data on avifauna in Southern Brazil. PLoS One. 2017;12: 1–27. 10.1371/journal.pone.0188819 29228053 PMC5724844

[34] Kocaman S, Gokceoglu C. Possible contributions of citizen science for landslide hazard assessment. Int Arch Photogramm Remote Sens Spat Inf Sci—ISPRS Arch. 2018;42: 295–300. 10.5194/isprs-archives-XLII-3-W4-295-2018

[35] Newman G, Zimmerman D, Crall AW, Laituri M, Graham J, Stapel L. User-friendly web mapping: Lessons from a citizen science website. Int J Geogr Inf Sci. 2010;24: 1851–1869. 10.1080/13658816.2010.490532

[36] Pennington C, Freeborough K, Dashwood C, Dijkstra T, Lawrie K. The National Landslide Database of Great Britain: Acquisition, communication and the role of social media. Geomorphology. Elsevier B.V.; 2015;249: 44–51. 10.1016/j.geomorph.2015.03.013

[37] Powell J, Nash G, Bell P. GeoExposures: Documenting temporary geological exposures in Great Britain through a citizen-science web site. Proc Geol Assoc. 2012;124: 638–647. 10.1016/j.pgeola.2012.04.004

[38] Kocaman S, Gokceoglu C. A CitSci app for landslide data collection. Landslides. Landslides; 2018; 1–5. 10.1007/s10346-018-1101-2

[39] Murthy YVNK, Raju PLN, Srivastav SK, Kumar P, Mitra D, Karnatak H, et al Capacity building for collecting primary data through crowdsourcing—An example of disaster affected Uttarakhand State (India). Int Arch Photogramm Remote Sens Spat Inf Sci—ISPRS Arch. 2014;XL–8: 1249–1252. 10.5194/isprsarchives-XL-8-1249-2014

[40] Paul JD, Buytaert W, Allen S, Ballesteros-Cánovas JA, Bhusal J, Cieslik K, et al Citizen science for hydrological risk reduction and resilience building. Wiley Interdiscip Rev Water. 2017;5: e1262 10.1002/wat2.1262

[41] Chu HJ, Chen YC. Crowdsourcing photograph locations for debris flow hot spot mapping. Nat Hazards. Springer Netherlands; 2018;90: 1259–1276. 10.1007/s11069-017-3098-6

[42] Choi CE, Cui Y, Zhou GGD. Utilizing crowdsourcing to enhance the mitigation and management of landslides. Landslides. Landslides; 2018;15: 1889–1899. 10.1007/s10346-018-1034-9

[43] Baum RL, Highland L, Lyttle PT, Fee JM, Martinez EM, Wald LA. “Report a Landslide” A Website to Engage the Public in Identifying Geologic Hazards. Landslide Sci a Safer Geoenvironment. 2014;1: 1–486. 10.1007/978-3-319-04999-1

[44] Samodra G, Chen G, Sartohadi J, Kasama K. Generating landslide inventory by participatory mapping: an example in Purwosari Area, Yogyakarta, Java. Geomorphology. Elsevier B.V.; 2018;306: 306–313. 10.1016/j.geomorph.2015.07.035

[45] U.S. Geological Survey. Catastrophic Landslides of the 20th Century—Worldwide [Internet]. [cited 26 Feb 2019]. Available: https://landslides.usgs.gov/learn/majorls.php

[46] Wikipedia. List of Landslides [Internet]. 2019 [cited 26 Feb 2019]. Available: https://en.wikipedia.org/wiki/List_of_landslides

[47] Cruden DM, Varnes DJ. Landslides: investigation and mitigation. Chapter 3-Landslide types and processes. Spec Rep—Natl Res Counc Transp Res Board. 1996;247: 36–75.

[48] U.S. Geological Survey. Landslide Types and Processes. In: Facts Sheet 2004–3072 [Internet]. 2004 pp. 1–4. Available: https://pubs.usgs.gov/fs/2004/3072/

[49] Juang CS. Help NASA Build the Largest Open Landslide Catalog with Landslide Reporter. In: SciStarter Blog: Citizen Science Projects, People, and Perspectives. 2018.

[50] Juang CS. Help NASA Build the Largest Open Landslide Catalog with Landslide Reporter. In: CitizenScience.gov [Internet]. 2018 [cited 28 Feb 2019]. Available: https://www.citizenscience.gov/2018/03/22/nasa-landslide-reporter-project/#

[51] Patel K. Help NASA Create the Largest Landslide Database. NASA Earth Observatory: Earth Matters Blog. Greenbelt; 18 4 2018 Available: https://earthobservatory.nasa.gov/blogs/earthmatters/2018/04/18/landslide-citizen-science/

[52] Petley DN. You can help compile the NASA landslide catalogue. In: AGU Blogosphere: The Landslide Blog [Internet]. 2018 [cited 28 Feb 2019]. Available: https://blogs.agu.org/landslideblog/2018/03/27/nasa-landslide-catalogue-1/

[53] EarthSky Voices. Help NASA create the world’s largest landslide database. EarthSky. 2018 Available: https://earthsky.org/earth/help-nasa-create-worlds-largest-landslide-database

[54] Patel K. Before and After the Kerala Floods. NASA Earth Observatory: Image of the Day. 2018 Available: https://earthobservatory.nasa.gov/images/92669/before-and-after-the-kerala-floods

[55] Causer T, Wallace V. Building a Volunteer Community: Results and Findings from Transcribe Bentham. Digit Humanit Q. 2012;6 Available: http://www.digitalhumanities.org/dhq/vol/6/2/000125/000125.html

[56] Gura T. Amateur Experts. Nature. 2013;496: 259–261. 10.1038/nj7444-259a 23586092

[57] Kirschbaum DB, Stanley T, Yatheendradas S. Modeling landslide susceptibility over large regions with fuzzy overlay. Landslides. Landslides; 2016;13: 485–496. 10.1007/s10346-015-0577-2

[58] Marc O, Stumpf A, Malet J, Gosset M, Uchida T, Chiang S. Towards a global database of rainfall-induced landslide inventories: first insights from past and new events. 2018; 1–28.

[59] Herrera G, Mateos RM, García-Davalillo JC, Grandjean G, Poyiadji E, Maftei R, et al Landslide databases in the Geological Surveys of Europe. Landslides. 2017; 1–21. 10.1007/s10346-017-0902-z

[60] Eveleigh A, Jennett C, Blandford A, Brohan P, Cox AL. Designing for Dabblers and Deterring Drop-Outs in Citizen Science. Proceedings of the SIGCHI Conference on Human Factors in Computing Systems. Toronto: ACM; 2014. pp. 2985–2994. 10.1145/2556288.2557262

[61] Bowser A, Hansen D, Preece J, He Y, Boston C, Hammock J. Gamifying citizen science. Proceedings of the companion publication of the 17th ACM conference on Computer supported cooperative work & social computing—CSCW Companion ‘14. 2014. pp. 137–140. 10.1145/2556420.2556502

[62] Bessette-Kirton EK, Coe JA, Godt JW, Kean JW, Rengers FK, Schulz WH, et al Preliminary Locations of Landslide Impacts from Hurricane Maria, Puerto Rico. In: U.S. Geological Survey data release [Internet]. 2017 [cited 27 Feb 2019]. 10.5066/F7JD4VRF

[63] West S, Pateman R. Recruiting and Retaining Participants in Citizen Science: What Can Be Learned from the Volunteering Literature? Citiz Sci Theory Pract. 2016;1: 1–10. 10.5334/cstp.8

