# Supplementary material for: Using citizen science to expand the global map of landslides: Introducing the Cooperative Open Online Landslide Repository (COOLR)
Source: PLoS One. 2019 Jul 3;14(7):e0218657. doi: 10.1371/journal.pone.0218657 (PMC6608936; doi:10.1371/journal.pone.0218657)
Supplement: S1 File — The detailed protocol used for checking all citizen science data contributed to the Cooperative Open Online Landslide Repository (COOLR) through the Landslide Reporter application as of February 2019. (PDF) [file pone.0218657.s001.pdf]

Using citizen science to expand the global map of landslides: Introducing the Cooperative Open Online Landslide Repository (COOLR)
Caroline S. Juang,[1,2*¶] Thomas A. Stanley,[1,3&] Dalia B. Kirschbaum[1&]

[1]Hydrological Sciences Laboratory, NASA Goddard Space Flight Center, Greenbelt, MD, USA.
[2]Hydrospheric and Biospheric Sciences, Science Systems and Applications, Inc., Lanham, MD, USA.
[3]Universities Space Research Association, Columbia, MD, USA.

*Corresponding author
Email: cjuang@alumni.harvard.edu

S1 File: Data Validation Protocol

Detailed here are parts of the protocol used for checking all citizen science data contributed to the Cooperative Open Online Landslide Repository (COOLR) through the Landslide Reporter application as of February 2019. **This process is subject to change as the COOLR project continues to improve.**

Checking for duplicates

The data from the Landslide Reporter Catalog (LRC) is overlaid with data of all other previously recorded events in COOLR in Esri ArcMap. COOLR data in the surrounding area, centered on the reported landslide event, are selected with the “Identify” tool. The distance from the reported landslide event to the farthest COOLR event in the selection is dependent on the location accuracy of the reported landslide event. The NASA reviewer compares the date of the reported event to the dates of the selected events from COOLR. If the date does not overlap with any of the events in COOLR and the event details do not match any of the other events in the selection, it is assumed to be a new event. If the date of the reported event matches or is very close in date to an event already in COOLR, the descriptions of the events are compared.

Checking for sufficient accuracy and detail

Sufficient accuracy and detail are checked by looking at each row of data entered in the database in Esri ArcMap. The criteria for checking each landslide report is as follows:

Sufficient accuracy:

1. *If the report links to a source*:

   a. Compare the landslide event’s location on the map to the location information given in the source. All given location details from the source must match the report. Check that the appropriate location accuracy is given.

b. Compare the details of the event, including the date, landslide category, trigger, fatalities, landslide setting, and other fields. All information must match either the information in the source or the photo given in the source.

2. *If the report is an in-person observation*:

a. Compare the landslide event's location on the map to the location information given in the location description. If the landslide event is old enough and at a scale that satellite imagery is available, compare the landslide location on the map to historical satellite imagery in Google Earth.

b. If a photo is included in the event report, use the photo to verify details of the event including the landslide category, landslide setting, and possibly landslide trigger.

Sufficient detail:

3. *If the report links to a source*:

a. Verify if all of the details from the source have been captured and put into the landslide event report in the fields that can be filled.

4\. *If the report is an in-person observation*:

a. Since details cannot be properly verified for accuracy, check that all fields in the report are filled out with as much information as possible. Enough detail must be written or filled in as possible so that it is possible to understand when, why, and how the landslide may have occurred, and optionally what the consequences were.

If the new citizen scientist-contributed report does not have sufficient detail or has inaccuracies, the reviewer makes corrections to the report based on the source linked in the report. If the source information is not in English, Google Translate (https://translate.google.com) is used to gather as much information as possible. If a report does not have source information and the report does not have any detail, the landslide report will be deleted.

Once a report is sufficiently accurate and detailed, the NASA reviewer copies the report from the inventory of submissions held for review to COOLR and then deletes the report from the inventory of submissions held for review. Data deleted will no longer appear when a citizen scientist accesses the "My Submissions" section in Landslide Reporter, and citizen scientists are not notified if their report has been approved or not at this point.
